# Functional and molecular characterization of the conserved Arabidopsis PUMILIO protein, APUM9

**DOI:** 10.1007/s11103-019-00853-7

**Published:** 2019-03-13

**Authors:** Tünde Nyikó, Andor Auber, Etienne Bucher

**Affiliations:** 1 Université d’Angers, UMR1345 Institut de Recherche en Horticulture et Semences (IRHS-INRA), 42 rue Georges Morel, 24, 49071 Beaucouzé, France; 2 Agricultural Biotechnology Institute, Szent-Györgyi Albert 4, Gödöllő, 2100 Hungary

**Keywords:** APUM9, PUMILIO/PUF protein, RNA-binding protein, Transposable element, Plant development and heat tolerance, ABA

## Abstract

**Key message:**

Here we demonstrate that the APUM9 RNA-binding protein and its co-factors play a role in mRNA destabilization and how this activity might regulate early plant development.

**Abstract:**

APUM9 is a conserved PUF RNA-binding protein (RBP) under complex transcriptional control mediated by a transposable element (TE) that restricts its expression in Arabidopsis. Currently, little is known about the functional and mechanistic details of the plant PUF regulatory system and the biological relevance of the TE-mediated repression of APUM9 in plant development and stress responses. By combining a range of transient assays, we show here, that APUM9 binding to target transcripts can trigger their rapid decay via its conserved C-terminal RNA-binding domain. APUM9 directly interacts with DCP2, the catalytic subunit of the decapping complex and DCP2 overexpression induces rapid decay of APUM9 targeted mRNAs. We show that APUM9 negatively regulates the expression of ABA signaling genes during seed imbibition, and thereby might contribute to the switch from dormant stage to seed germination. By contrast, strong TE-mediated repression of *APUM9* is important for normal plant growth in the later developmental stages. Finally, APUM9 overexpression plants show slightly enhanced heat tolerance suggesting that TE-mediated control of APUM9, might have a role not only in embryonic development, but also in plant adaptation to heat stress conditions.

**Electronic supplementary material:**

The online version of this article (10.1007/s11103-019-00853-7) contains supplementary material, which is available to authorized users.

## Introduction

The Arabidopsis PUMILIO9 (APUM9) protein is a member of the highly conserved family of PUF RNA-binding proteins (referred also as PUMILIO proteins) found in all eukaryotes. In animals and yeast PUF proteins are negative regulators of gene expression that bind with high specificity to canonical sequences within the 3′UTR of their target mRNAs by means of their RNA-binding domain (RBD) thereby stimulating transcript decay or translational repression (Wharton et al. 1998; Deng et al. 2008; Quenault et al. 2011; Friend et al. 2012). The conserved RNA-binding domain (RBD or PUF domain) of classical PUF proteins comprises eight imperfect tandem repeats (PUF-repeats), in which each of the three highly conserved amino acids are responsible for binding to a single ribonucleotide base of the target transcript (Lu et al. 2009). Thus they typically recognize an eight nucleotide long canonical sequence, 5′-UGUANAUA, with an “UGUA” core, referred to as PUMILIO response element (PRE) (Wang et al. 2013; Hogan et al. 2015; Prasad et al. 2016). However, atypical interactions to non-canonical RNA sequences were also identified in yeast and plants (Porter et al. 2015; Zhang and Muench 2015). Once bound, they can recruit Argonaute proteins to attenuate translational elongation or more frequently the CCR4-NOT-POP2 deadenylase complex and/or decapping enzymes to destabilize target transcripts (Wickens et al. 2002; Goldstrohm et al. 2006; Chritton and Wickens 2010; Friend et al. 2012; Van Etten et al. 2012; Weidmann et al. 2014; Prasad et al. 2016). Removal of the cap and/or the poly(A) tail has been proposed to initiate the degradation of mRNA either by XRN1-mediated 5′exonucleolytic decay or by 3′exosome decay, or both (Parker and Song 2004). Although conserved interactions between PUF proteins and POP2 deadenylase subunit are well described from yeast to mammals the precise decay mechanism(s) remains to be unraveled (Goldstrohm et al. 2006).

The conserved PUF domain was also shown to be involved in protein–protein interactions that can strongly influence the activity and specificity of PUF proteins. Protein partners can tune PUF activity in a tissue or development-specific manner leading to altered RNA-binding affinity or change of affinity to target mRNAs (Wickens et al. 2002; Weidmann et al. 2016). A well-known, classical example is the localized collaboration of the *Drosophila* PUMILIO protein with Nanos zinc finger protein to allow abdomen formation by repressing hunchback mRNA in the posterior pole of the embryo (Lehmann and Nüsslein-Volhard 1987; Weidmann and Goldstrohm 2012). In mammals, a conserved long noncoding RNA (lncRNA), NORAD, can serve as a molecular decoy and control the activity of PUM1–2 PUF proteins to preserve genome stability (Lee et al. 2016; Tichon et al. 2016).

PUF proteins are essential developmental regulators with an ancestral conserved role in stem cell maintenance but species specific roles like embryonic patterning, germline switch, neuronal and mitochondrial functions were also described (Campbell et al. 2012; Crittenden et al. 2002; Kaye et al. 2009; Lander et al. 2012; Lin and Spradling 1997; Salvetti et al. 2005; Spassov and Jurecic 2003; Spradling et al. 2001; Vessey et al. 2010). Putative PREs can be detected in a large fraction of genes (approximately 7–11%) in all studied model organisms (Galgano et al. 2008; Gerber et al. 2006; Kershner and Kimble 2010; Morris et al. 2008; Wilinski et al. 2015). Perturbed PUF activity cause severe developmental phenotypes and defects in mitotic cell division and stem cell control (Wreden et al. 1997).

In Arabidopsis, 26 PUF proteins, called APUMs (for “Arabidopsis PUMILIO”), were identified many of which represent examples of gene duplication events (Tam et al. 2010). They show considerable variability in their PUF-repeat number, position and amino acid sequence. Over half of the APUMs (APUM1–15) possess all the eight PUF repeats and only six of them (APUM1–6) show conservation at the key amino acids responsible for target binding. Due to this great variability they can recognize additional non-cognate sequences beyond the canonical PRE motifs (Zhang and Muench 2015). It is still unknown whether plant APUMs suppress their target through translational inhibition or by mRNA destabilization. Functional data for only a few APUMs have been reported. Indirect evidence revealed that similar to other eukaryotic PUF proteins, APUM1–6 might be involved in stem cell control and differentiation regulating the key plant developmental factors FASCIATA-2, WUSCHEL, CLAVATA-1 and ZWILLE/PINHEAD in the shoot apical meristem (Francischini and Quaggio 2009). APUM5 has a role in plant defense mechanisms inhibiting cucumber mosaic virus (CMV) replication and regulating abiotic stress response genes (Huh et al. 2012; Huh and Paek 2014). APUM23 and APUM24 show nucleolar localization and appear to be required for pre-ribosomal RNA processing. APUM23 plays role in shoot and root development, while APUM24 is essential for normal cell division patterning during early embryogenesis (Abbasi et al. 2011; Huang et al. 2014; Shanmugam et al. 2017). Overall, these data suggest that APUM proteins might act in a similar manner in plants like in other organisms and that they may have important roles in plant development. However, the molecular and functional details of plant PUF-regulatory system are still poorly understood.

APUM9 (AT1G35730) possesses all eight PUF repeats and displays high similarity to the yeast PUF4 protein in key amino acid positions (Francischini and Quaggio 2009). APUM9 transcripts are very low in almost all tissues except during the first half of seed maturation and in imbibed seeds (Francischini and Quaggio 2009; Xiang et al. 2014). This strong tissue specificity of *APUM9* is the result of the presence of *ROMANIAT5*, a copia-like retrotransposon (RTE) within its promoter region, that brings this gene under a complex epigenetic control. The synergistic effect of two epigenetic factors, MOM1 (MORPHEUS’ MOLECULE1) and NRPE1 (NUCLEAR RNA POLYMERASE E1), are required for strong *APUM9* repression (Hristova et al. 2015; Yokthongwattana et al. 2010). High tissue specificity of *APUM9* promoter activity can be tuned depending on the combination of *trans* factors being expressed in different tissues raising the possibility of tissue specific epigenetic code in plants (Yokthongwattana et al. 2010). For instance, HDA6 (HISTONE DEACETYLASE 6) defective plants release *APUM9* repression only in young developing leaves supporting the notion that RTE-mediated control of *APUM9* might be important for normal plant growth (Hristova et al. 2015). Furthermore, the heat-responsive RTE located in the promoter regions promotes partial release of the *APUM9* promoter under heat stress conditions suggesting that *APUM9* might have role not only in plant development but also in heat tolerance (Pietzenuk et al. 2016). These observations suggest that APUM9 regulation might be particularly complex however the exact role of this protein is still not unraveled.

The aim of this work is to understand how APUM9 regulates mRNA stability and its physiological importance. We show here, that APUM9 binding triggers rapid decay of target mRNAs by directly interacting with the main degradation complexes. Based on our results we propose a role for APUM9 in early development, promoting the switch from dormant stage to seed germination in imbibed seeds by modulating ABA signaling. Overexpression of APUM9 results in abnormal leaf development, late flowering phenotype and slightly enhanced heat tolerance suggesting that RTE-mediated control of *APUM9* transcription might have a role not only in plant development but also in plant adaptation to heat stress conditions.

## Results

### APUM9 binding induces target mRNA degradation via its conserved C-terminal RBD

**Fig. 1:**
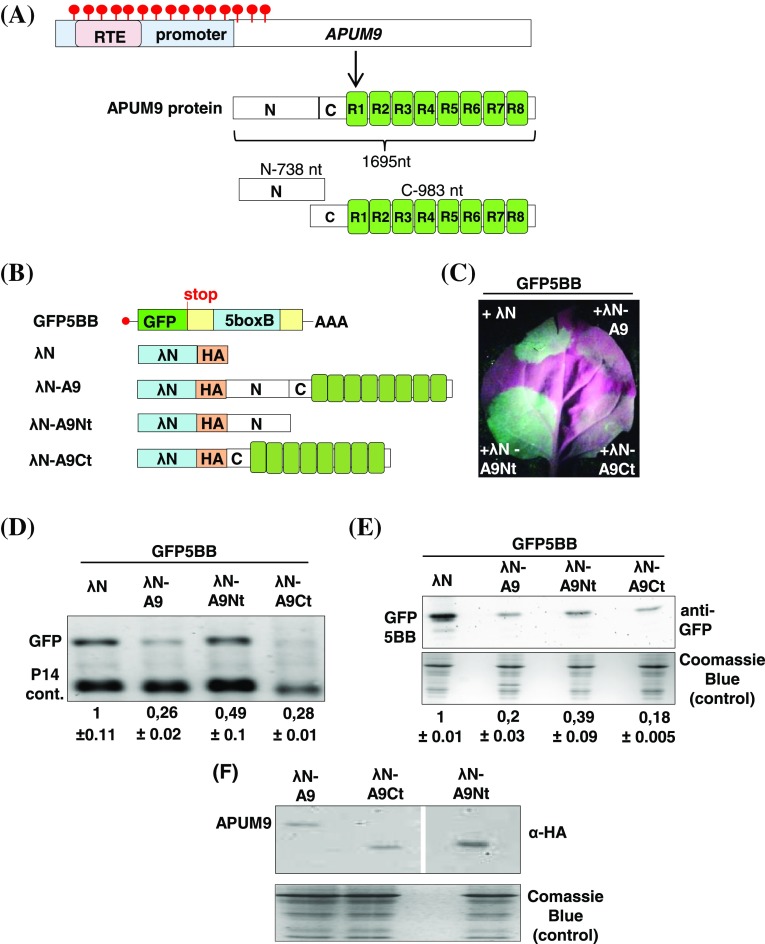
APUM9 binding triggers target mRNA degradation. **a** Schematic, non-proportional representation of *Arabidopsis APUM9* gene and protein structure. Red circles show DNA methylation induced by the RTE. The eight conserved RNA binding repeats are shown as green rectangles (R1–R8). N and C terminal protein domains of APUM9 are labelled as N and C. **b** Schematic, non-proportional representation of the constructs used for tethering assay. GFP5BB reporter is shown as transcript, while λN tethering constructs will act as proteins. **c** APUM9 induces mRNA decay. To test the effect of APUM9 binding on target mRNA, GFP5BB reporter plus P14 silencing supressor were co-expressed in *N. benthamiana* leaves with full-length, N-, or C-terminal APUM9 tethering constructs (λN-A9, λN-A9Nt, λN-A9Ct). As control, GFP5BB + P14 leaves were infiltrated with λN. P14 was co-infiltrated in each mix to suppress RNA silencing and served as an infiltration control for RNA gel-blot assays. Photo and RNA-, protein samples were taken at 3 days post infiltration (d.p.i). Photo was taken under UV light, thus the non-infiltrated parts of the leaf are red due to the autofluorescence of chlorophyll, while the GFP expressing agroinfiltrated patches show green fluorescence. **d** RNA gel blot was hybridized with GFP and P14 probes. To quantify RNA samples, at each lane the signal of the reporter mRNA (GFP probe) was normalized to the corresponding P14 signal (GFP/P14 signal). Mean values were calculated from three independent samples (*n* = 3). To estimate effect of APUM9 on reporter mRNA stability, the GFP/P14 ratio of the GFP5BB + λN sample was taken as 1 and the GFP/P14 ratio of λN-A9, λN-A9Nt, λN-A9Ct co-infiltrated samples are shown relative to it (± shows standard deviation, SD). Note that co-expression of GFP5BB with λN-A9 and λN-A9Ct constructs leads to weak fluorescence and low GFP/P14 signal. **e** GFP Western blot further confirmed that GFP5BB expression is significantly lower in λN-A9 and λN-A9Ct co-infiltrated samples compared to the control. GFP protein signals were normalized to the corresponding comassie blue stained total protein level as described above for RNA blot. **f** To confirm the expression of APUM9 fusion proteins (λN-A9, λN-A9Nt, λN-A9Ct), they were immunoprecipitated with HA antibody from protein extracts derived from the corresponding agroinfiltrated leaves

The conserved C-terminal RBD of PUF proteins recognize an eight nucleotide long RNA motif (PRE) within the 3′UTR of target mRNAs resulting in the destabilization or repression of the translation of the bound transcript (Supplementary Fig. 1). Currently, little is known about the requirements for PREs or the effect of plant APUM binding to target mRNA have on RNA stability or translation. To investigate this in more detail, we used a tethering system to artificially attach APUM9 to a reporter mRNA in order to identify the effect APUM9 binding to target transcripts has. *Nicotiana benthamiana* (*N. benthamiana*) plants were agroinfiltrated with two plasmids, one that expresses a λN-APUM9 (λN-A9) fusion protein, and a second one, that expresses a GFP reporter mRNA harboring five copies of BoxB (5BB) sequences within its 3′UTR (GFP5BB) (test mix) (Fig. 1a, b and Supplementary Fig. 2a). The λN-peptide strongly and sequence-specifically binds to the short BB RNA segment (Baron-Benhamou et al. 2004), therefore the λN-A9 fusion protein is artificially bound (tethered) to the GFP5BB reporter transcript. λN-peptide without APUM9 fusion or the APUM9 without λN fusion (A9) was co-infiltrated with GFP5BB as negative control sample (control mix) (Fig. 1c and Supplementary Fig. 2b). If APUM9 tethering leads to mRNA destabilization, co-infiltration of λN-A9 but not λN or A9 should result in a decrease of the transcript and protein levels of GFP5BB, while translational repression by APUM9 should only influence GFP5BB protein accumulation. In order to analyze whether the non-conserved N-terminal or the highly conserved C-terminal part of APUM9 is required for target repression, the λN-A9Nt and λN-A9Ct constructs, expressing only the N-, or C-terminal APUM9 domains were also co-infiltrated with GFP5BB (Fig. 1b). The P14 silencing suppressor was co-infiltrated with each sample to prevent agroinfiltration-induced transgene silencing and to serve as an internal control for northern blot normalizations (Merai et al. 2005). For further details about the role of P14 see Fig. 1.

3 days post infiltration (3 d.p.i.) GFP fluorescence and mRNA levels were dramatically reduced in λN-A9 and λN-A9Ct tethered samples suggesting that APUM9 induces rapid degradation of the target mRNA (Fig. 1c, d). Western blot assays using GFP antibody further confirmed that λN-A9 and λN-A9Ct significantly reduced the expression of the GFP reporter (Fig. 1e). Co-infiltration of the λN-A9Nt weakly influence while λN and untethered APUM9 (A9) negative controls had no effect on GFP mRNA and protein level (Fig. 1c–e and Supplementary Fig. 2b, c). λN-A9, λN-A9Nt, λN-A9Ct fusion-proteins and A9 negative control accumulated to comparable levels (Fig. 1f and Supplementary Fig. 2e). Additional tethering assays using GUS5BB reporter to measure GUS mRNA and GUS activity levels further support our findings that APUM9 suppresses target mRNAs by decreasing the transcript level (Supplementary Fig. 3a–c). The conserved C-terminal domain of APUM9 can efficiently trigger mRNA degradation, while the non-conserved N-terminal had a weaker effect on target expression (Fig. 1d and Supplementary Fig. 3a–c). These data suggest that, similar to other eukaryotes, the conserved C-terminal RBD of APUM9 can stimulate rapid degradation of bound mRNA. However we cannot exclude that the N-terminal part might also contribute to target mRNA destabilization (see “Discussion” section for details).

### Overexpression of DCP2 enhances APUM9-mediated mRNA destabilization

In human cells, PUF proteins promote deadenylation-dependent mRNA decay by recruiting the POP2 catalytic subunit of the CCR4-NOT deadenylase complex to their RNA substrate (Van Etten et al. 2012). Alternatively, co-recruitement of decapping factors (DCP1,2) via POP2 interaction can also facilitate mRNA degradation (Blewett and Goldstrohm 2012; Quenault et al. 2011). Catalytic subunits of Arabidopsis decapping and deadenylase complex, DCP2 (AT5G13570) and CAF1a (AT3G44260) respectively, and their catalytically inactive dominant negative (DN) forms have already been described previously (Gunawardana et al. 2008; Liang et al. 2009). The analogous highly conserved *N. benthamiana* NbDCP2 and NbCAF1a wild-type and DN forms were also identified and provided from Daniel Silhavy’s lab prior to publication (See Data S1-Description of cloning for details).

**Fig. 2:**
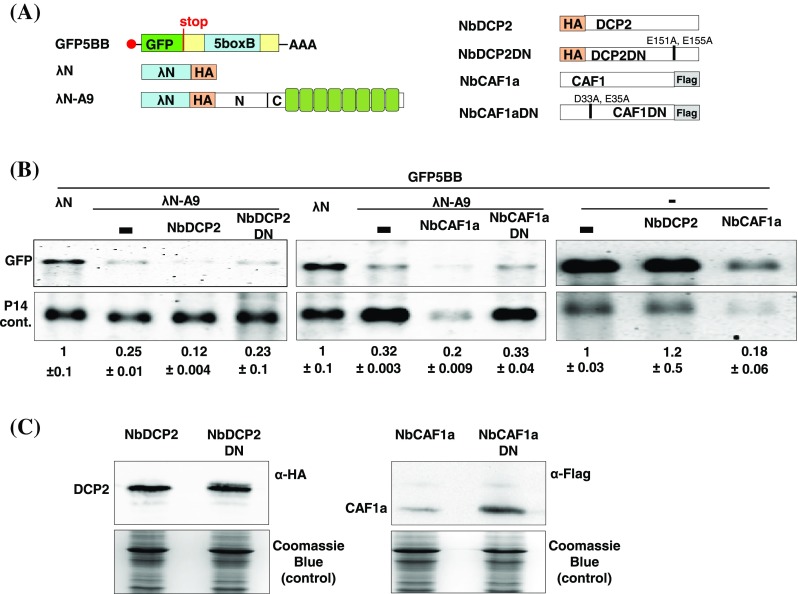
DCP2 overexpression strongly enhances APUM9-mediated mRNA decay. **a** Schematic, non-proportional representation of the agroinfiltrated constructs. **b***N. benthamiana* leaves were infiltrated with GFP5BB target construct + P14 + λN as negative or GFP5BB + P14 + λN-A9 as positive controls. To test whether DCP2 and/or CAF1a has any role in APUM9-mediated RNA degradation, NbDCP2, NbDCP2DN, NbCAF1a or NbCAF1aDN constructs were added into the GFP5BB + P14 + λN-A9 agroinfiltration mixes. GFP5BB + P14 without λN-A9 were co-infiltrated with NbDCP2 or NbCAF1a to test the specificity of DCP2 and CAF1a in APUM9-mediated decay. RNA levels were quantified as described in Fig. 1**c** The expression of HA tagged NbDCP2, NbDCP2DN and FLAG tagged NbCAF1a and NbCAF1aDN constructs were confirmed by western blot

To test if these complexes participate in APUM9-mediated target degradation, we transiently repressed decapping or deadenylase activity in agroinfiltrated *N. benthamiana* leaves by co-infiltrating the DN versions of NbDCP2 or NbCAF1a (NbDCP2DN, NbCAF1aDN) including the GFP5BB reporter with or without λN-A9 (Fig. 2a). We hypothesized that if DCP2 or CAF1a are involved in APUM9-mediated mRNA decay, co-infiltration of the DN form would lead to a less efficient mRNA degradation in λN-A9 samples. Wild-type NbDCP2 and NbCAF1a were co-infiltrated as controls (Fig. 2a). Surprisingly, DN forms had no effect on GFP mRNA level while overexpression of either wild-type NbDCP2 or NbCAF1a enhanced GFP mRNA degradation in λN-A9 co-infiltrated samples (Fig. 2b left and central panel). Notably, CAF1a co-expression reduced the GFP mRNA level also in control samples without λN-A9 (Fig. 2b right panel). In contrast, DCP2 selectively reduced GFP mRNA levels only in λN-A9 samples suggesting that as long as CAF1a influences the rates of general mRNA turnover DCP2 can specifically enhance APUM9-mediated mRNA decay. This conclusion is supported by the finding that the level of the P14 internal control was also significantly lower in CAF1a co-infiltrated samples. Comparable levels of NbDCP2 and NbCAF1a protein accumulation was confirmed from all constructs by western blot (Fig. 2c). Accordingly, we hypothesize that APUM9 bound transcripts can be degraded via a decapping-dependent exonucleolytic pathway but deadenylation-dependent decay might also be involved. This redundancy could explain why we do not see any effect resulting from DN infiltration.

### XRN4 and SKI2 are not essential for APUM9-mediated mRNA decay

**Fig. 3:**
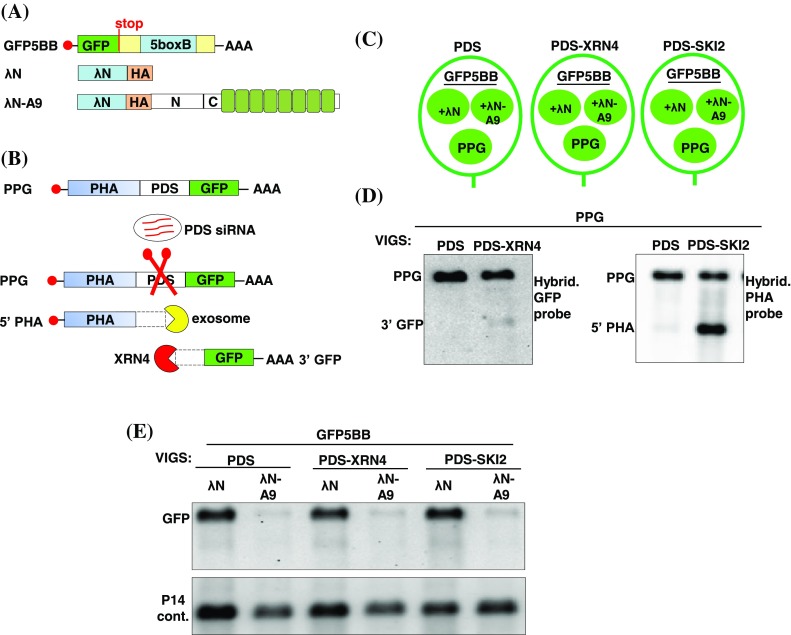
XRN4 and SKI2 are not essential for APUM9-mediated mRNA degradation. **a** Schematic, non-proportional representation of the constructs used. **b** The PPG VIGS sensor transcript is cleaved by PDS siRNAs generated form VIGS vectors. 5′PHA and 3′GFP cleavage fragments of PPG are quickly degraded by exosome or XRN4 exonucleases respectively. **c** Leaves of PDS-silenced (PDS) negative control and PDS + XRN4 or PDS + SKI2 silenced test *N. benthamina* plants (PDS–XRN4 and PDS–SKI2, respectively) were agroinfiltrated with P14 + PPG silencing sensor construct or with GFP5BB + P14 + λN, GFP5BB + P14 + λN-A9 tethering test constructs. **d** RNA samples from PPG infiltrated leaf patches were hybridized with GFP or PHA probes. Accumulation of the 3′GFP and 5′PHA cleavage products of PPG suggests the efficiency of XRN4 and SKI2 silencing. **e** To monitor whether XRN4 and/or SKI2 deficiency could stabilize the APUM9 targeted mRNAs, RNA samples from GFP5BB + P14 + λN and GFP5BB + P14 + λN-A9 infiltrated PDS-, PDS–XRN4 and PDS–SKI2 leaf patches were hybridized with GFP

Our results suggested that tethering of APUM9 to target mRNAs initiated transcript degradation mainly by activating decapping. Here, we wanted to test if deadenylation may also contribute to APUM9-mediated mRNA decay. Following decapping and deadenylation in plants, the XRN4 exoribonuclease and SKI-exosome cytoplasmic exonuclease complex have been shown to be required for cytoplasmic 5′–3′ and 3′–5′ mRNA degradation, respectively (Parker and Song 2004). SKI2 RNA-helicase, is an essential cofactor for cytoplasmic exosome activity (Branscheid et al. 2015). To unravel these late steps of mRNA degradation we combined transient virus induced gene silencing (VIGS) system with our tethering assay (for details see also Experimental Procedures) (Kerenyi et al. 2008). Test-, and control tethering mixes and the PHA–PDS–GFP (PPG) silencing test construct were infiltrated into PDS-silenced negative control-, PDS–XRN4-, and PDS–SKI2-silenced test leaves (Fig. 3a, b, c). The PPG fusion construct was used to test silencing efficiency in the infiltrated *N. benthamiana* leaves. Briefly, in all three silenced plants, PDS siRNAs generated from VIGS vectors cleaved the PPG transcript in the PDS linker region (Fig. 3b). 5′PHA and 3′GFP cleavage fragments of PPG are quickly degraded and could not be detected in the PDS-silenced control plants (Fig. 3d). In contrast, accumulation of 5′PHA and 3′GFP cleavage products in PDS–SKI2 and PDS–XRN4-silenced plants, respectively confirm that the SKI2 and XRN4 silencing was effective (Fig. 3d). We have postulated that if the degradation of APUM9 bound transcripts are accomplished by exonucleolityc XRN4-, or exosome activities, mRNA destabilization induced by λN-A9 tethering would be less efficient in XRN4-, and/or SKI2 silenced leaves. By contrast GFP5BB mRNA was degraded with comparable efficiency in the PDS-, PDS-XRN4 and PDS–SKI2 silenced plants suggesting that XRN4 and exosome are not essential for exonucleolytic decay of APUM9-bound mRNA (Fig. 3e). Alternatively, degradation could also be initiated with an endonucleolytic cleavage of the GFP5BB target. In this case the 3′ GFP cleavage fragment should be detectable in XRN4-silenced leaves, because no other 5′–3′cytoplasmic exonuclease was found in plants. However, we could not detect the 3′cleavage product of GFP5BB transcripts in XRN4-silenced leaves indicating that APUM9-mediated degradation is not initiated by an endonucleolytic cleavage or that cleavage occurs in the 3′UTR region of the GFP5BB reporter transcript, close to the APUM9 binding site. In the latter case the 3′ cleavage fragment is not detectable with our GFP probe.

Based on these results we conclude that XRN4 and SKI2 are not essential or might act redundantly in APUM9- mediated target degradation.

### APUM9 associates with the catalytic subunit of the plant decapping complex

**Fig. 4:**
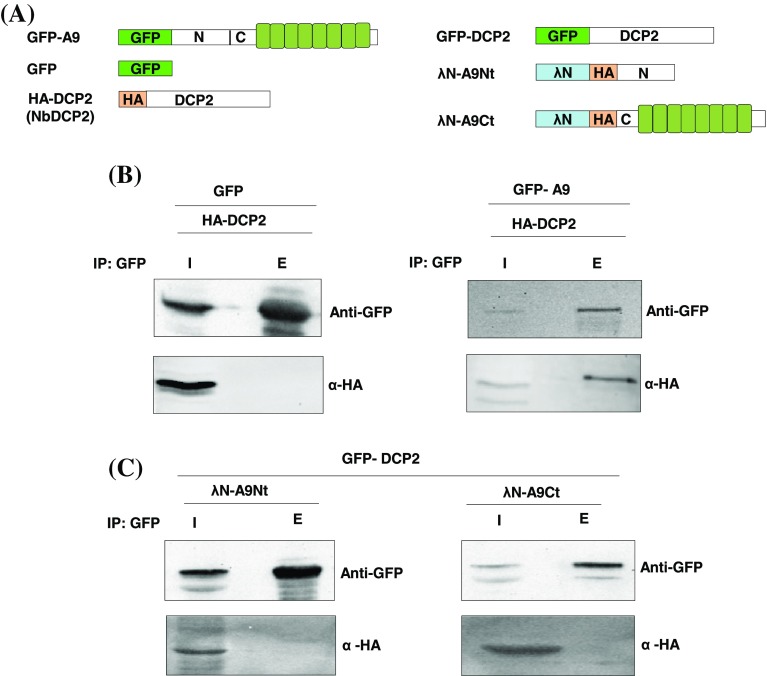
APUM9 is associated with the decapping complex through DCP2. **a** Schematic, non-proportional representation of the constructs used. **b***N. benthamiana* leaves were infiltrated with GFP and HA tagged DCP2 (HA–DCP2) as negative control and GFP–APUM9 fusion construct (GFP–A9) with HA tagged DCP2 (HA–DCP2) as test. 3 d.p.i. proteins were extracted and GFP co-immunoprecipitation was carried out. Input (I) and elutes of precipitate (E) were analyzed by western blotting. **c** GFP co-immunoprecipitation of HA- tagged A9Nt and A9Ct deletion mutants (λN-A9Nt, λN-A9Ct) with GFP–DCP2

PUMILIO proteins recruit multiple deadenylases and decapping factors to repress their target mRNAs in animals (Van Etten et al. 2012). To analyze the association of APUM9 with this enzyme in plants, GFP–APUM9 fusion protein (GFP–A9) was co-expressed with HA-tagged NbDCP2 (HA–DCP2) in *N. benthamiana* leaves and GFP–A9 was immunoprecipitated (IP) using anti-GFP antibodies coupled to agarose beads (Fig. 4a). Our results show that DCP2 strongly accumulated with GFP–A9 but not with the GFP negative control protein, thus we conclude that APUM9 directly interacts with DCP2 (Fig. 4b).

The highly conserved C-terminal RBD is sufficient to interact with POP2 and DCP decay enzymes in all studied eukaryotes. Since the λN-A9Ct construct triggered degradation more efficiently in our tethering assay we wanted to test whether in plants like in other eukaryotes, the C-terminal part of APUM9 might be involved in protein–protein interactions. To demonstrate this, we studied the binding capacity of HA-tagged APUM9 N-terminal and C-terminal domains (λN-A9Nt, λN-A9Ct) with GFP–DCP2 fusion protein, by GFP co-immunoprecipitation. Unexpectedly, both λN-A9Nt and λN-A9Ct failed to immunoprecipitate with GFP–DCP2 suggesting that, unlike yeasts and animals, the presence of full-length APUM9 may be essential for efficient interaction with DCP2 (Fig. 4a, c). Since the *N. benthamiana* PUMILIO9 was not identified yet and we could only detect putative *N. benthamiana* homologs with incomplete sequence information, we used the Arabidopsis APUM9 for the IP experiment (Supplementary Fig. 1). However, the subunits of the eukaryotic decapping complex are highly conserved, thus we assume that the detected interaction between Arabidopsis APUM9 and *N. benthamiana* DCP2 in our heterologous system reflects in vivo interactions of these proteins. Based on these results we conclude that DCP2 is a direct binding partner of APUM9.

### APUM9 plays a role in seed dormancy and TE-mediated repression of *APUM9* might be required for normal plant growth

The TE insertion in the promoter region contributes to tissue-specific expression of *APUM9*, restricted to the seeds and dehiscence zone of siliques (Fig. 1a and Supplementary Fig. 4a) (Hristova et al. 2015). Alterations in APUM9 expression strongly correlate with changes in seed dormancy level, indicating that APUM9 might have a role in early embryonic development (Xiang et al. 2014). However, the exact function of APUM9 is still unknown.

**Fig. 5:**
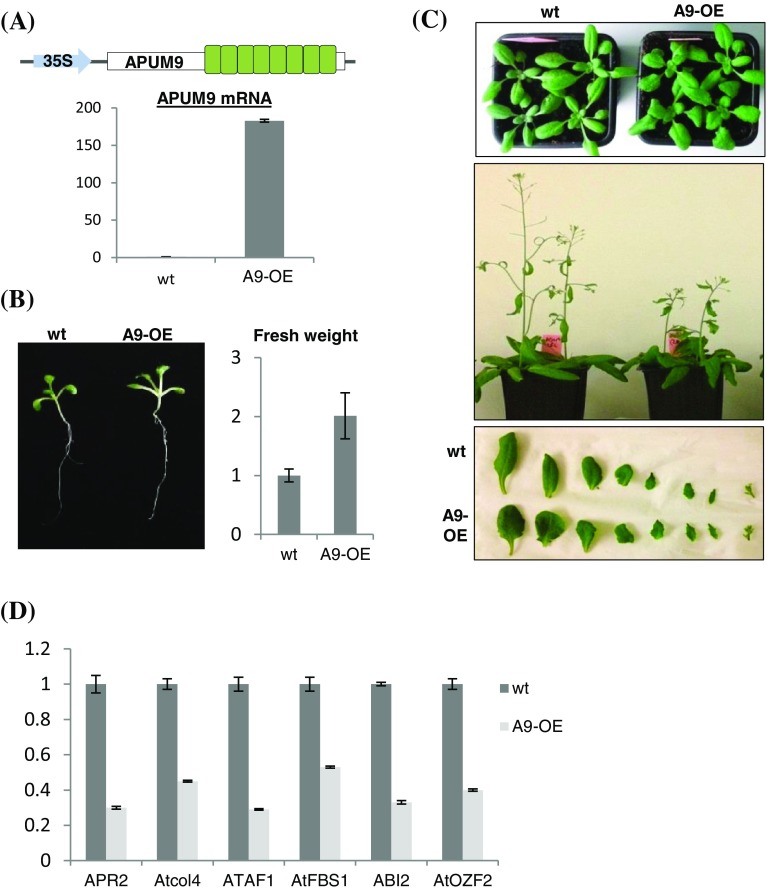
APUM9 is involved in early plant development. **a** qRT-PCR analysis of APUM9 transcript levels in Col-0 wild-type and APUM9 overexpression (A9-OE) transgenic *Arabidopsis* leaves. The expression values were normalized using UBIQUITIN as control, n = 3 biological replicates. **b** Fresh weight of Col-0 wild-type and A9-OE *Arabidopsis* plants were measured. Pictures show the analyzed 1 week old seedlings. Bars represent the mean of fresh weight measurement from 15 seedlings. Error bars indicate standard deviation (SD) of the mean. **c** Top and bottom pictures show the leaf morphology phenotype of 3 weeks old plants. All leaves of representative Col-0 wild-type and A9-OE transgenic plants were compared. Middle panel, late flowering phenotype of 4 weeks old Col-0 wild-type and A9-OE plants. **d** RNA-seq and qRT-PCR assays were conducted to compare the transcriptome profile of Col-0 wild-type and A9-OE imbibed seeds. The same RNA samples were used for both assays. The six down-regulated putative APUM9 targets were verified by qRT-PCR analysis. The expression values were normalized using UBIQUITIN as control, n = 3 biological replicates

In order to better understand the role of APUM9 in plant development, we first studied APUM9 insertion mutants (SALK_135897, SALK_028481, GK-152E12) but we could not detect any phenotypical consequence of APUM9 deficiency. This can be explained by redundancy with *APUM10* (*AT1G35750*), a duplicated gene pair of *APUM9*, located within 10 kb on the same chromosome and displaying a similar expression pattern (Abbasi et al. 2011; Tam et al. 2010). To further assess the effect of perturbed APUM9 level on plant development, we have generated APUM9 overexpression Arabidopsis lines (A9-OE), expressing APUM9 from the strong 35S constitutive promoter (Fig. 5a). Interestingly, A9-OE seedlings show enhanced growth in the early developmental phase (Fig. 5b) however APUM9 excess in later developmental stages resulted in an abnormal rounded leaf shape and late flowering phenotype (Fig. 5c). These results support the assumption that APUM9 might be involved in early plant development (Xiang et al. 2014), whereas transcriptional repression of APUM9 is required for normal growth in later developmental stages.

Increased expression of APUM9 has been shown to result in reduced seed dormancy in imbibed seeds (Xiang et al. 2014). To better understand the role of APUM9 in these early developmental stages, we compared the transcriptomes of imbibed seeds from wild-type and A9-OE lines to identify the potential target transcripts for APUM9. Because APUM9 facilitates the degradation of its target mRNAs we expected that transcripts directly targeted by APUM9 will show reduced expression in A9-OE line relative to the wild-type control. Notably, six significantly down-regulated transcripts (AT1G62180, AT5G24930, AT1G01720, AT1G61340, AT5G57050 and AT4G29190) can be observed from which five are involved in ABA signal transduction and induced by different abiotic stresses (Table 1). ATOZF2 (AT4G29190), ATAF1 (AT1G01720), ATFBS1 (AT1G61340) are positive-, while ABI2 (AT5G57050) and AtCol4 (AT5G24930) are negative regulators of ABA signaling pathway (Gonzalez et al. 2017; Huang et al. 2012; Jensen et al. 2013; Kim et al. 2016; Min et al. 2015) (Table 1). ABA is known to be involved in seed dormancy (Rodriguez et al. 2009). Indeed most of these down-regulated ABA genes were linked to seed dormancy and germination thus we hypothesize that APUM9 might regulate seed dormancy through the ABA signaling pathway (Table 1) (see “Discussion” section for details). The sixth down-regulated gene APR2 (AT1G62180) 5′ adenylylphosphosulfate reductase plays a role in plant growth and development, regulating the biosynthesis of sulfur-containing amino acids and might be responsible for abnormal leaf morphology and late flowering phenotype of A9-OE plants (Chao et al. 2014) (Fig. 5c; Table 1). RNA-seq results of all six transcripts were independently confirmed by real time-PCR (Fig. 5d).

**Table 1:** Down-regulated genes in A9-OE seeds that had been imbibed for 6 h

AGI	log2FoldChange	p Value	Description	Localisation	Involved in ABA	Function
AT1G62180	− 2.24	4.60E-09	5′adenylylphosphosulfate reductase (**APR2**), *positive regulator of ABI2*	Chloroplast	–	Biosynthesis of sulfur-containing amino acids (plant growth and developement)
AT5G24930	− 1.84	6.31E-06	Costans-like4 (**AtCol4**), zing finger transcription factor, *negative regulator of ABI2*	Nucleus	Yes (salt-,osmotic- and dehydration stress)	Salt stress resistance during *seed germination* and early development, enhance *seed dormancy* during abiotic stress conditions via ABA
AT1G01720	− 1.76	4.67E-05	ARABIDOPSIS NAC DOMAIN CONTAINING PROTEIN 2 (**ATAF1**), transcription factor, *positive regulator of ABI2*	Nucleus	Yes (wounding, salt-, drougth-, cold- stress)	*Germination and seedling development*
AT1G61340	− 1,65	3,56E-05	F-BOX STRESS INDUCED 1 (**AtFBS1**), F box family protein, *positive regulator of ABI2*	Nucleus	Yes (wounding, salt-, osmotic-, cold- stress)	Protein ubiquitination
AT5G57050	− 1.65	3.83E-05	ABA INSENSITIVE 2 (**ABI2**), protein phosphatase 2C (PP2C), *negative regulator of ABA*	Plasma membrane	Yes (heat stress)	Negative regulator of ABA, *reduced seed dormancy* during abiotic stress
AT4G29190	− 1.48	2.66E-03	OXIDATION-RELATED ZINC FINGER 2 (**AtOZF2**), transcription factor, *negative regulator of ABI2*	Nucleus, Plasma membrane	Yes (Salt- oxidative stress)	Salt and oxidative tolerance during *germination and early development*

### APUM9 overexpression promotes tolerance to ambient temperature stress

APUM9 repression is partially released during heat-stress conditions due to the HREs (heat responsive elements) located in the LTR of *ROMANIAT5* TE located in the promoter region (Pietzenuk et al. 2016). The A9-OE line grows significantly better at constant elevated (28 °C) temperature compared to wild-type (Supplementary Fig. 4b). To investigate if APUM9 activation in heat-stressed plants is physiologically relevant 7-day-old wild-type and A9-OE seedlings were exposed to 43 °C heat-shock for different time courses (Benamar et al. 2013). We could not observe any difference in heat tolerance between wild-type and A9-OE (Supplementary Fig. 4c). We thus conclude that APUM9 has no important role in plant heat stress response and it might only confer a limited/basal tolerance to smaller fluctuations in temperature.

## Discussion

In this study, we provide insights into the molecular mechanism of the so far poorly understood plant PUF-regulatory system.

### Conserved and novel aspects of plant PUF regulatory pathways

PUF proteins generally, act as repressors at the transcript level. Direct conserved interaction of the PUF RBD with deadenylation and decapping complex proteins promote mRNA destabilization or translation inhibition. However cap-, and polyA independent repressions were also described in *yeast* and mammals though the precise mechanisms remains unknown (Chritton and Wickens 2011; Van Etten et al. 2012). Our transient tethering assays demonstrate that plant APUM9 binding decreases the stability of target mRNAs (Fig. 1b–f, Supplementary Fig. 2b–d and Supplementary Fig. 3b–c). The conserved RBD of PUF proteins are known to physically interact with the POP2 subunit of the CCR4-NOT deadenylase complex or DCP2 decapping factor and activate mRNA degradation (Goldstrohm et al. 2006). Consistently, overexpression of both, the plant DCP2 factor and CAF1a deadenylase complex subunit, strongly enhanced APUM9 tethering induced target decay (Fig. 2b). Indeed, we have found that the C-terminal RBD of APUM9 triggers rapid decay of target mRNA similar to full-length APUM9, while the non-conserved N-terminal domain exhibits weaker repression (Fig. 1c–e and Supplementary Fig. 3b, c). Unexpectedly, only full-length APUM9 can interact with DCP2, suggesting that regions outside the RBD might also be involved to stabilize the interactions of APUM9 with its protein and/or mRNA partners (Fig. 4b, c). We postulate that, similar to yeast Puf2P, the N-terminal part of APUM9 might be crucial for stable mRNP complex formation (Porter et al. 2015), an important step we masked using an artificial tethering system (in the tethering experiment APUM9 does not need the N-terminal region for complex formation or for RNA binding). The N-terminal part might also have additional functions that stabilize APUM9–DCP2 protein interaction. Although we didn’t detect domains or motifs in the N-terminal region of APUM9, we could not exclude the possibility of unrecognized plant-specific domains and functions. Alternatively, tethering of the C-terminal RBD might induce alternative decapping-independent degradation pathways.

We could not detect a direct interaction between APUM9 and CAF1a suggesting that unlike in animals, plant APUM9 connect to the deadenylase complex via other subunits or they only form an RNA-dependent transient complex that we could not detect in our IP conditions. Our observations that DCP2 specifically enhances APUM9-mediated mRNA decay, while CAF1a stimulates the efficiency of overall mRNA turnover suggest that APUM9 bound mRNAs are predominantly degraded by a decapping-dependent mechanism (Fig. 2b). It is also likely that multiple degradation pathways cooperate to rapidly remove the APUM9 “marked” mRNAs and to avoid trapping APUM9 complexes on target transcripts.

The late degradation steps following decapping and deadenylation of PUF bound mRNAs are not well described. In yeast, XRN1 and exosome complex are the major cytoplasmic 5′–3′ and 3′–5′ exoribonucleases, but deletion of the SKI2, XRN1 exonucleases did not influence PUF5p repression (Goldstrohm et al. 2006). Similarly, APUM9-mediated repression was unaffected by depletion of cytoplasmic plant XRN4 exonuclease and SKI2 exosome complex proteins suggesting that these enzymes are not essential for APUM9 mediated target degradation or that they play a redundant role in mRNA degradation. (Fig. 3d, e). Since we could not detect the 3′cleavage products of GFP5BB transcripts in XRN4 silenced leaves, we can exclude the possibility that APUM9 by itself initiates endonucleolytic cleavage of the target transcript.

In animals, PUF-proteins selectively bind to a conserved 8 nt long PRE in the 3′UTR of target mRNAs. However, plant PUF-proteins can also recognize various non-cognate sequences beyond the canonical PRE motifs. APUM1–6 can bind the canonical PRE bound by *Drosophila* and human PUMILIOs, while the binding sequence of APUM23 is a non-canonical 10 nt long RNA motif with an atypical UUGA core (Francischini and Quaggio 2009; Zhang and Muench 2015). Indeed, APUM24 binds RNA with no apparent specificity (Shanmugam et al. 2017). In order to investigate whether similar to other eukaryotic PUF proteins, APUM9 acts through binding to the 3′UTR of their target mRNAs we selected three APUM9 down-regulated genes (AT1G62180, AT5G24930, AT1G61340) and cloned their 3′UTRs after GFP reporter (GFP180, GFP930, GFP340). GFP reporters were co-infiltrated with λN-A9 as test and λN as negative control (Supplementary Fig. 6b). In the absence of 5BB sequence, λN-A9 could destabilize the GFP constructs only if it binds to their 3′UTRs. Our results show that the fluorescence of GFP180 and GFP930 were significantly reduced in λN-A9-, compared to λN co-infiltrated samples. qPCR measurements further confirm that APUM9 might regulate the expression of GFP180 and GFP930 reporters through binding to their 3′UTR (Supplementary Fig. 6b). However comparing the 3′UTRs of these APUM9 down-regulated transcripts we could not identify a consensus sequence motif. We thus hypothesize that similar to APUM24, APUM9 recognizes diverse atypical plant specific core sequences or bind to mRNA in a sequence-independent manner. Further analyses are required to properly define the binding specificity of APUM9.

Taken together our data suggest that APUM9-mediated mRNA repression operates similarly to other eukaryotes via decapping and/or deadenylation dependent exonucleolytic mRNA decay pathways. We propose that similar to APUM9, some APUMs may also act by destabilizing mRNAs with different tissue or sequence speicificity, however this aspect still needs to be tested. Since the ability of PUF proteins to interact with different protein partners allows the assembly of many distinct PUF protein complexes, which might activate different mRNA repression mechanisms, we cannot generally exclude the possibility of APUM-mediated translation repression in plants.

### Biological significance of APUM9 in plant development and heat-tolerance

In plants the APUM family consist of a greater number of members than in any other species, up to 25 APUMs were identified in Arabidopsis (Tam et al. 2010). This large number of highly similar copies could be indicative of an ongoing selective pressure leading to the evolution of novel and highly specific PUF-regulatory networks in plants. Functional data suggest that several APUMs preserved their ancestral role in stem cell control but acquired plant-specific functions like CMV virus inhibition and abiotic stress response were also described (Francischini and Quaggio 2009; Huh et al. 2012).

APUM9 is strongly repressed in almost all tissues except seeds, most likely due to TE-mediated transcriptional repression (Fig. S4a). Partial release of APUM9 during seed imbibition negatively correlates with seed dormancy level, however the molecular background of this regulation remains to be further investigated (Xiang et al. 2014). In contrast to the previous predictions that APUM9 might regulate seed dormancy by affecting the translation efficiency of mRNAs stored in seeds (Xiang et al. 2014), our tethering assay suggests that APUM9 acts through mRNA destabilization (Fig. 1d). Our transcriptome analysis from imbibed seeds reveals only a a limited number of significantly downregulated genes in A9-OE lines, most of them involved in ABA signaling (Fig. 5d; Table 1). Beside its important role in abiotic stress responses ABA also plays a central role in the induction and maintenance of seed dormancy and inhibits the transition from embryonic stage to germination (Rodriguez et al. 2009) (Supplementary Fig. 5a). Consequently, ABA accumulates in seeds during their development reaching high levels during seed maturation and in dry seeds which then decreases during seed imbibition (Rodriguez-Gacio Mdel et al. 2009). A direct relationship between APUM9 release and the ABA content was not tested yet. Here we propose a speculative model for how APUM9 could influence seed dormancy level by destabilizing ABA signaling genes. Upregulation of APUM9 in imbibed seeds might decrease the efficiency of ABA signaling by inducing rapid degradation of the respective ABA transcripts. Decreased ABA sensitivity in turn enhances the transition from dormant stage to seed germination (Supplementary Fig. 5b). Notably, the rice PUMILIO1 protein was recently found to interact with dormancy associated proteins which further support our model (Sugiharti et al. 2013). Furthermore, APUM24 was recently shown to be required for normal cell division during early embryogenesis by regulating the proper flow of auxin in plant embryos (Shanmugam et al. 2017). Our transcriptome analysis was performed on plants strongly overexpressing APUM9 (A9-OE). It is possible that under natural conditions, partial release of APUM9 might only cause minimal alterations in ABA sensitivity and may thus only have a minor contribution to the switch from dormant stage to seed germination. Additionally, APUM9 acts redundantly and/or co-operate with its duplicated gene pair APUM10 and with APUM11 thus co-silencing of these three genes could only enhance seed dormancy level (Xiang et al. 2014). Interestingly, APUM10 and APUM11 expression is also very low in all plant tissues similar to APUM9 (Supplementary Fig. 6a). However, in contrast to APUM9, the expression of APUM10 and APUM11 were not released in wild-type imbibed seeds which partially contradicts our hypothesis that they might act redundantly in seed dormancy regulation (Supplementary Fig. 6a). Albeit APUM10 was never directly linked to seed development and an increase of APUM11 in imbibed seeds was so far only detected in a mutant Arabidopsis background (Xiang et al. 2014). Based on these observations we presume that APUM9 might have a primary role in seed dormancy, while in certain cases APUM11 and/or APUM10 could contribute to this regulation. Based on these preliminary data further exploration is important to unravel the link between ABA level and APUM9 release in imbibed seeds. Transgenic lines moderately expressing APUM9 from its own promoter without the TE insertion would allow a more detailed study of the importance of APUM9 in seed dormancy fluctuations.

Our finding that APUM9 overexpression results in late flowering and leaf morphology defects suggests that tissue-specific repression of APUM9 may be important for normal plant development (Fig. 5c). Indeed, a slightly increased level of APUM9 transcripts in wild-type leaves and flowers coincides with previous qPCR measurements made by Xiang et al. 2014 and it might also support our findings that tissue-specific repression of APUM9 may have role in normal leaf morphogenesis and early plant development (Fig. 5 and Supplementary Fig. 6a). We propose that the highly abundant transgenic APUM9 protein in A9-OE lines may strongly influence the stability of transcripts involved in flowering and leaf development. Further experiments are required to better characterize the fine-grained regulation of target transcripts degradation by APUM9 in different tissues. Given the high degree of sequence similarity and overlapping functions of APUMs, it is likely that they are essential for proper plant development, but future investigations are needed to evaluate this question.

The *ROMANIAT5* TE insertion is a unique feature of Arabidopsis *APUM9* promoter, absent from other Arabidopsis and plant species (Pietzenuk et al. 2016). Heat-responsiveness of the TE promotes partial release of *APUM9* expression under heat stress conditions, hence we proposed that this novel feature of APUM9 might contribute to advanced heat tolerance in Arabidopsis. However, we could only detect an enhanced tolerance to an elevated ambient temperature but not to a harsh heat stress (Supplementary Fig. 4b, c). Therefore, we conclude that APUM9 might only confer a limited/basal heat tolerance to small increases in temperature.

Through our work on APUM9, we provide new insights into the molecular mechanisms and function of plant PUMILIO proteins and offer an important basis for future studies investigating the exact role of APUM9 in seed dormancy and the specificity of APUM9–RNA and APUM9–protein binding.

## Materials and methods

### Plasmid constructs

The details of clonings and other constructs are described in Data S1. The primers that were used for cloning are listed in Table S1.

### Plant materials and growth conditions

*Arabidopsis APUM9* insertion mutants, *apum9-2* (SALK_135897), *apum9-3* (GK-152E12-013134) and *apum9-1 (SALK_028441)* were obtained from the Arabidopsis Biological Resource Center (ABRC), Nottingham Arabidopsis Stock Centre **(**NASC) and Wim J.J. Soppe respectively. Homozygous T-DNA insertion lines were identified with PCR using gene-specific primers and T-DNA primers (Table S2). *Arabidopsis* plants were grown in a growth chamber at 22/20 °C under 16/8 h light/dark conditions.

For fresh weight measurement 2 weeks old wild-type and A9OE Arabidopsis plants were used, grown on 0,5X MS media at 22°/20 °C or constant 28° under 16/8 h light/dark conditions.

For heat shock treatments, freshly harvested seeds were sown into a six-well plate, containing 6 ml EVIAN mineral water in each well and grown at 22/20 °C under 16/8 h light/dark conditions with moderate shaking. Seven days old seedlings were subjected to heat-shock at 43 °C heat stress for different time-course (30 min, 45 min, 1 h, 1 h 10 min, 1 h 20 min, 1 h 30 min) using a water bath incubator. Heat sensitivity became visible 7 days after stress (Benamar et al. 2013).

Late flowering was determined by counting the total number of leaves, excluding the cotyledons, since there is a close correlation between leaf number and flowering time.

Floral dip transformation was used to generate transgenic APUM9 overexpression *Arabidopsis* line (A9-OE).

Agroinfiltrated and VIGS-treated *N. benthamiana* plants were grown in Panasonic MLR-352H-PE chambers at 24 °C/22 °C with 16 h light/8 h dark.

### Agroinfiltration based transient gene expression assays

Agroinfiltration assay were performed as described (Kertesz et al. 2006). Bin61S binary vectors were introduced into the C58C1 *Agrobacterium tumefaciens* strain. Wild-type, 3 weeks-old *N. benthamiana* leaves were agroinfiltrated with a mixture of different agrobacterial cultures (OD600 of each culture was 0.4, except P14 that was 0.2). RNA and protein samples were collected 3 days after agroinfiltration (3 d.p.i). GFP fluorescence was detected 3.d.p.i by using Nightsea Stereo Microscope Flourescence Adapter (Nightsea, http://www.nightsea.com).

### VIGS agroinfiltration experiment

To transiently silence *PDS, XRN4* and *SKI2* genes in *N. benthamiana*, a 600–700 nucleotide long segment from these genes were incorporated into a *Tobacco Rattle Virus* (TRV) VIGS vector and 3 weeks-old *N. benthamiana* plants were infected with the recombinant *TRV* VIGS vectors. TRV infection induces antiviral RNA silencing response, that will specifically inactivate the host genes that are homologous to the incorporated sequence. PDS (phytoene desaturase) control silencing leads to photobleaching (leaf whitening), which alleviates the monitoring of silencing. Thus 10–12 days after infection, when the upper leaves started to bleach (indicating that PDS silencing was efficient and suggesting that the silencing of the gene of interest is also effective), leaves below the bleaching ones were agroinfiltrated with a mixture of tethering constructs. Technical details of VIGS methodology were previously described (Kerenyi et al. 2008; Ratcliff et al. 2001).

### RNA gel blot analysis

RNA samples isolated from agroinfiltrated leaves at 3.d.p.i were separated in formaldehyde-MAE containing denaturing agarose gel and blotted to nylon membrane (Roche). Hybridization and detection were conducted according to the “North2South™ Complete Biotin Random Prime Labeling and Detection Kit” (Thermofisher) instructions. We have used ChemiDoc XRS + imaging system for detection and ImageLab 5.0 software (Bio-Rad, 1708265) to analyse the mRNA blot.

### GUS–NAN activity measurement

Total protein lysates were prepared at 3 d.p.i. in lysis buffer containing 50 mM phosphate buffer pH 7, 10 mM β-mercaptoethanol, 10 mM disodium EDTA pH 8, 0.1% sodium laurylsarcosine, 0.1% Triton X-100, 1 mM PMSF. NAN and GUS activity assays were carried out in 96-well-black assay plates (VWR) in 50 µl assay buffer (50 mM phosphate buffer, pH 7; 10 mM β-mercaptoethanol) containing either 0.4 mM MUN or MUG (Sigma) as substrates, respectively, for NAN and GUS. Reactions were incubated at 37 °C for 30 min, and terminated by adding an equal volume of 0.4 M Na_2_CO_3_. Methylumbelliferone (MU) fluorescence was measured (excitation at 355 nm, emission at 460 nm) using a FLUOstar Omega multi-mode microplate reader (BMG Labtech). GUS activity was normalized to the corresponding NAN control signal.

### Protein-immunoprecipitation (IP) and Western blot

Protein extraction and IP was carried out as described by Baumberger and Baulcombe (Baumberger and Baulcombe 2005, except G-25 separation was omitted) at 3 d.p.i. ANTI-HA AFFINITY MATRIX (Roche) and ANTI-GFP-TRAP®_A BEADS (Chromoteck) were used for IPs. Samples were separated in SDS–PAGE, blotted onto AMERSHAM PROTRAN MEMBRANE (Sigma) and hybridized with monoclonal ANTI-HA-PEROXIDASE (Roche), rabbit polyclonal ANTI-GFP (Thermofisher) and ANTI-FLAG (Sigma) antibodies. Chemiluminescent protein detections were conducted according to the instructions (ECL WESTERN BLOTTING SUBSTRATE (Promega) for HA and CDPstar (Sigma) for GFP and Flag). Western blots were scanned with ChemiDoc XRS + System and analyzed with ImageLab 5.0 software (Bio-Rad). For Coomassie blue staining protein gels were incubated in 0.25% Coomassie Blue R-250, for overnight and destained for 2–4 h in 40% MeOH, 10% HOAC destaining solution.

### Quantitative RT-PCR Analysis

Total RNA from 100 mg of fresh leaves, siliques and seeds of *Arabidopsis* plants was isolated using TRIZOL® REAGENT. After DNAse treatement, 200 ng of RNA was used for cDNA synthesis (M-MLV REVERSE TRANSCRIPTASE, Promega). qPCR was carried out with IQ SYBR® GREEN SUPERMIX (Bio-Rad) in a PTC-200 DNA ENGINE THERMAL CYCLER (Bio-Rad) machine. qRT-PCR-primers are listed in Table S3.

### RNA seq and transcriptome analyse

Total RNA was extracted from 100 mg of wild-type and A9-OE imbibed Arabidopsis seeds, 6 h after imbibition following the Rapid Trisol based two step method by Ling Meng and Lewis Feldman (Meng and Feldman 2010). Differential gene expression was assessed by DESeq2 using two biological replicates for each line (Love et al. 2014).

## Electronic supplementary material

Below is the link to the electronic supplementary material.

Supplementary material 1 (PPTX 2664 KB)—Supplementary Figure 1–6

Supplementary material 2 (DOCX 40 KB)—Data S1

Supplementary material 3 (XLSX 3630 KB)—Data S2
