# Supplementary material for: Functional and molecular characterization of the conserved Arabidopsis PUMILIO protein, APUM9
Source: Plant Mol Biol. 2019 Mar 13;100(1):199–214. doi: 10.1007/s11103-019-00853-7 (PMC6513901; doi:10.1007/s11103-019-00853-7)
Supplement: Supplementary file 1 — Supplementary material 1 (PPTX 2664 KB)—Supplementary Figure 1–6 [file 11103_2019_853_MOESM1_ESM.pptx]

## Slide 1
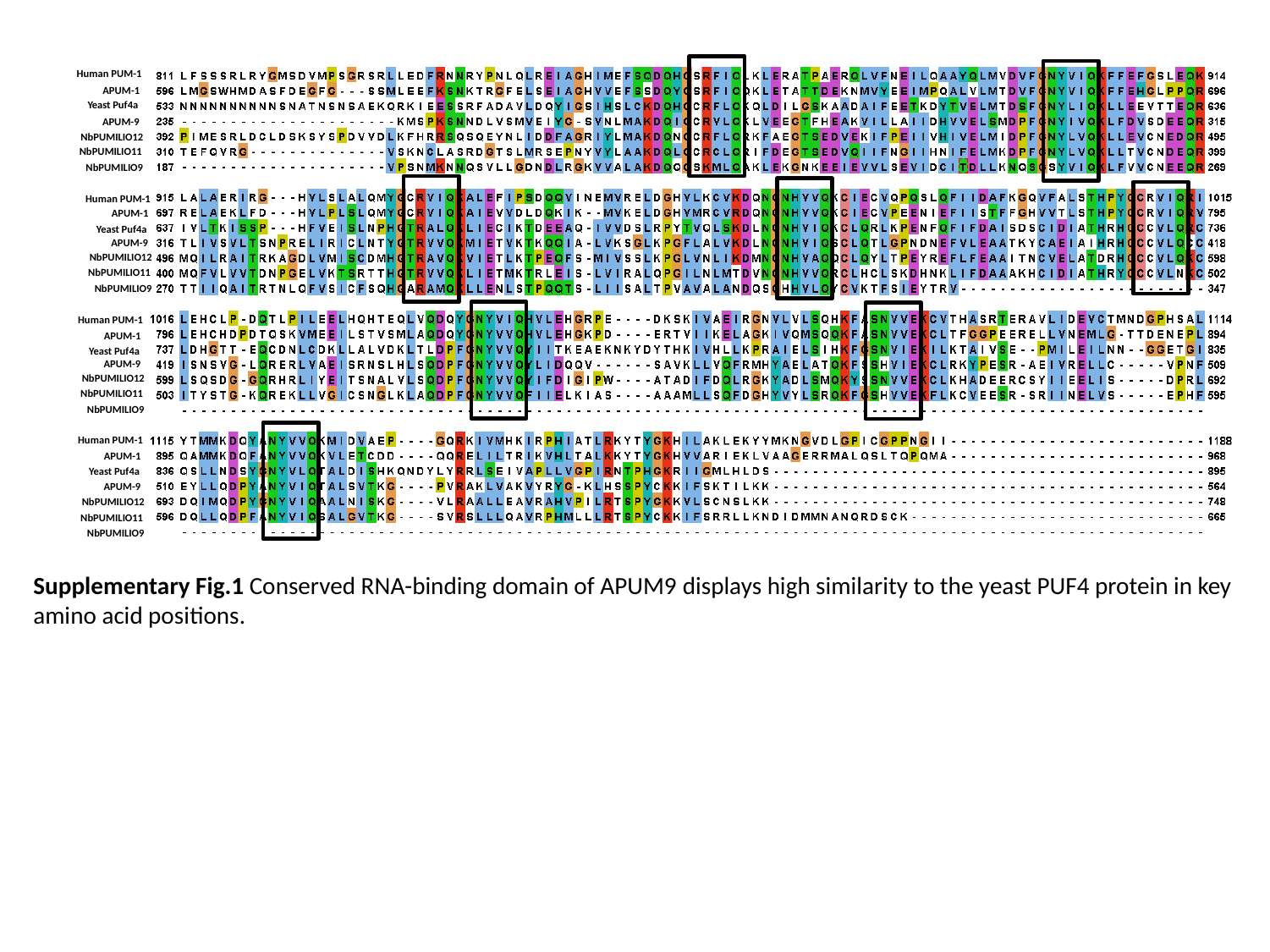

Human PUM-1
APUM-1
Yeast Puf4a
APUM-9
NbPUMILIO12
NbPUMILIO11
NbPUMILIO9
Human PUM-1
APUM-1
Yeast Puf4a
APUM-9
NbPUMILIO12
NbPUMILIO11
NbPUMILIO9
Human PUM-1
APUM-1
Yeast Puf4a
APUM-9
NbPUMILIO12
NbPUMILIO11
NbPUMILIO9
Human PUM-1
APUM-1
Yeast Puf4a
APUM-9
NbPUMILIO12
NbPUMILIO11
NbPUMILIO9
Supplementary Fig.1 Conserved RNA-binding domain of APUM9 displays high similarity to the yeast PUF4 protein in key amino acid positions.

## Slide 2
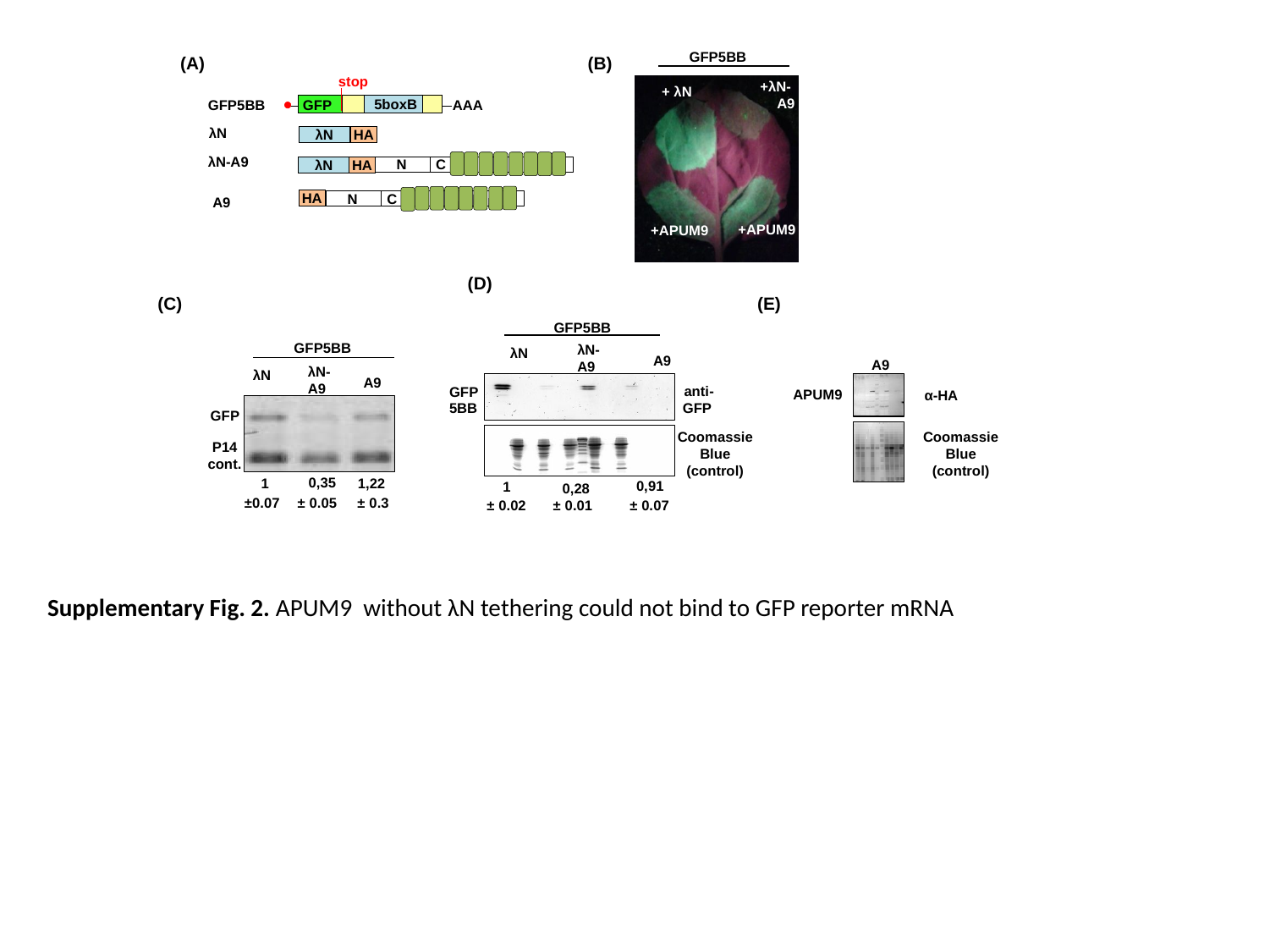

GFP5BB
(a)
(b)
stop
+λN-
A9
+ λN
GFP5BB
GFP
AAA
5boxB
λN
λN
HA
λN-A9
N
C
HA
λN
C
N
A9
HA
+APUM9
+APUM9
(d)
(c)
(e)
GFP5BB
A9
GFP5BB
A9
APUM9
α-HA
Coomassie
Blue
(control)
λN-
A9
λN
A9
λN-
A9
λN
anti-
GFP
GFP
5BB
GFP
Coomassie
Blue
(control)
P14
cont.
0,35
1
1,22
0,91
1
0,28
±0.07
± 0.3
± 0.05
± 0.02
± 0.01
± 0.07
Supplementary Fig. 2. APUM9 without λN tethering could not bind to GFP reporter mRNA

## Slide 3
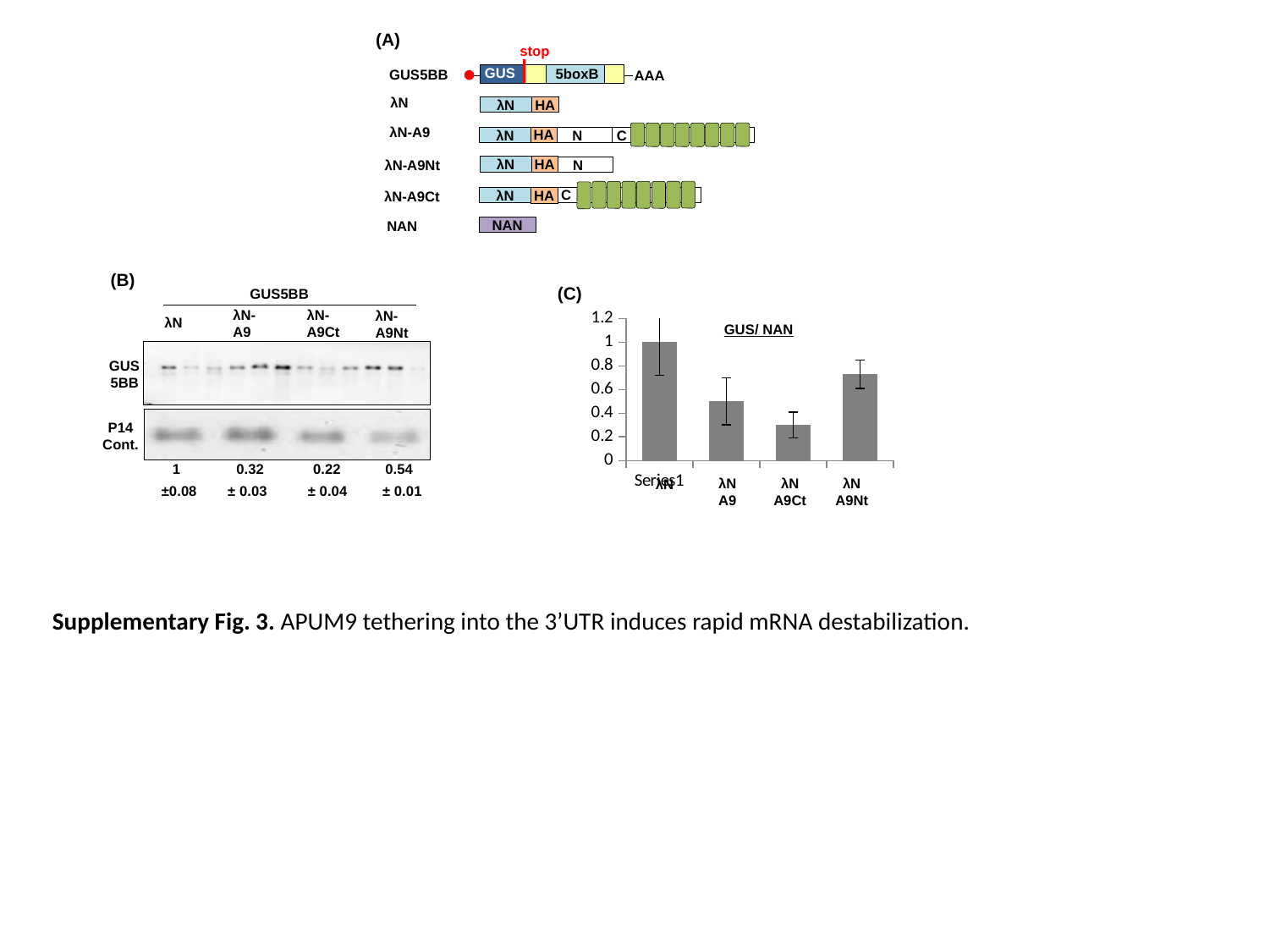

(a)
stop
GUS
GUS5BB
AAA
5boxB
λN
λN
HA
λN-A9
C
N
HA
λN
N
λN-A9Nt
λN
HA
C
λN-A9Ct
λN
HA
NAN
NAN
(b)
(c)
GUS5BB
λN-
A9Ct
λN-
A9
λN-
A9Nt
### Chart
| Category | |
|---|---|
| | 1.0 |
| | 0.5 |
| | 0.3000000000000003 |
| | 0.7300000000000006 |λN
GUS/ NAN
GUS
5BB
P14
Cont.
0.54
1
0.32
0.22
λN
A9Ct
λN
A9
λN
A9Nt
λN
±0.08
± 0.03
± 0.04
± 0.01
Supplementary Fig. 3. APUM9 tethering into the 3’UTR induces rapid mRNA destabilization.

## Slide 4
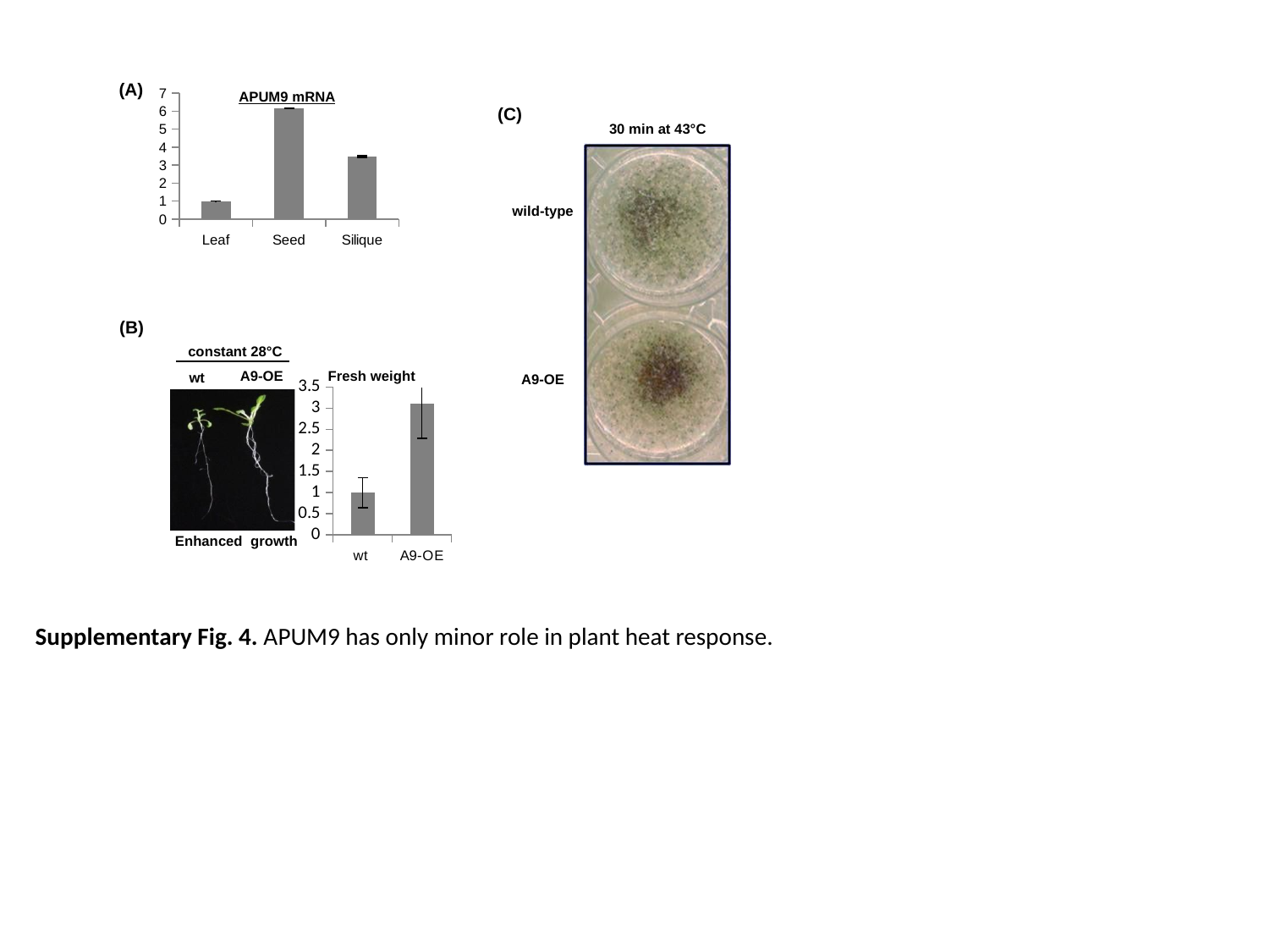

(a)
### Chart
| Category | |
|---|---|
| Leaf | 1.0 |
| Seed | 6.14 |
| Silique | 3.48 |APUM9 mRNA
(c)
30 min at 43°C
wild-type
(b)
constant 28°C
Fresh weight
A9-OE
wt
A9-OE
### Chart
| Category | |
|---|---|
| wt | 1.0 |
| A9-OE | 3.1 |
Enhanced growth
Supplementary Fig. 4. APUM9 has only minor role in plant heat response.

## Slide 5
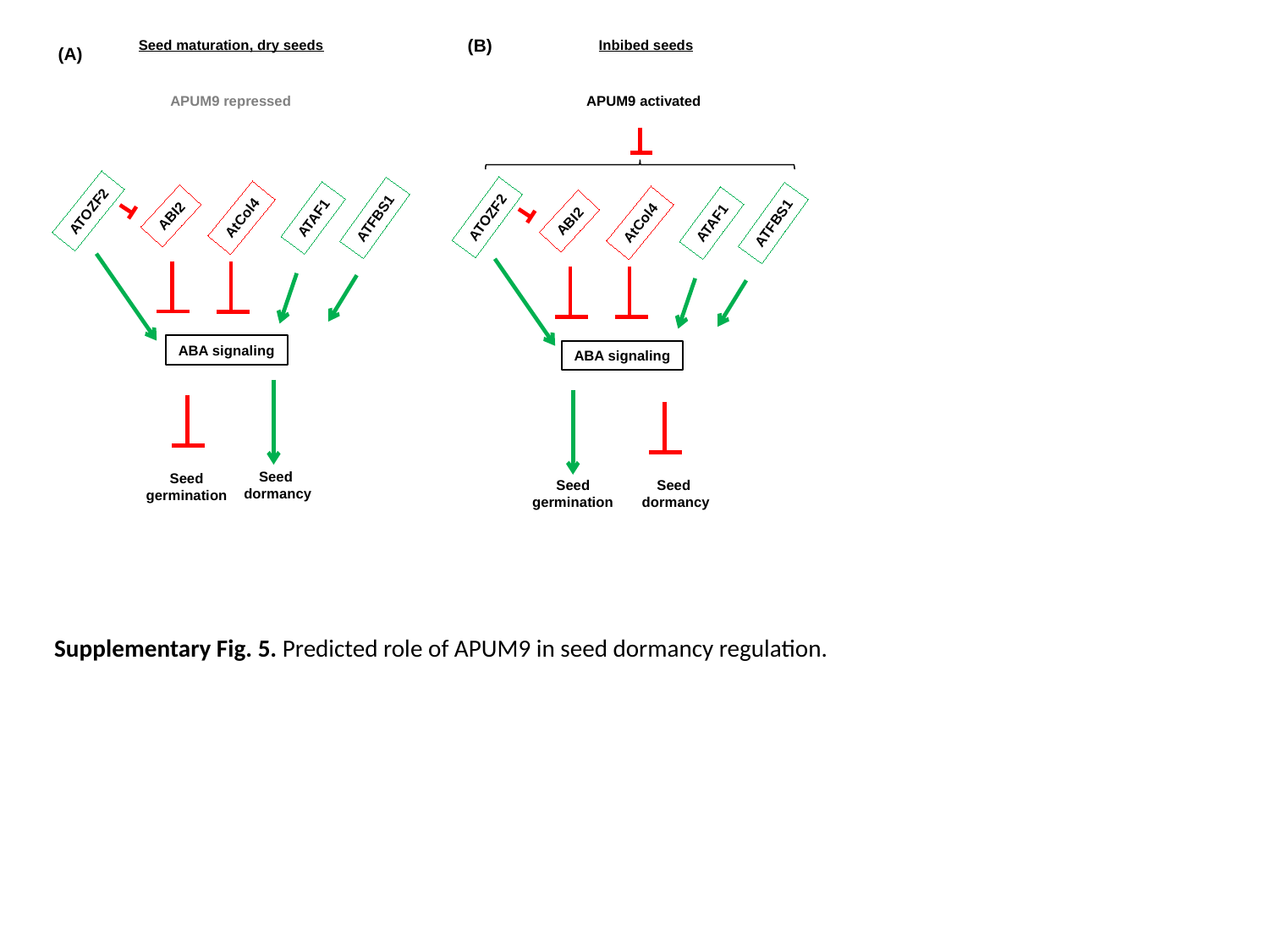

(b)
Seed maturation, dry seeds
Inbibed seeds
(a)
APUM9 repressed
APUM9 activated
ATOZF2
ABI2
AtCol4
ATAF1
ATFBS1
ATOZF2
ABI2
AtCol4
ATAF1
ATFBS1
ABA signaling
ABA signaling
Seed
dormancy
Seed
germination
Seed
dormancy
Seed
germination
Supplementary Fig. 5. Predicted role of APUM9 in seed dormancy regulation.

## Slide 6
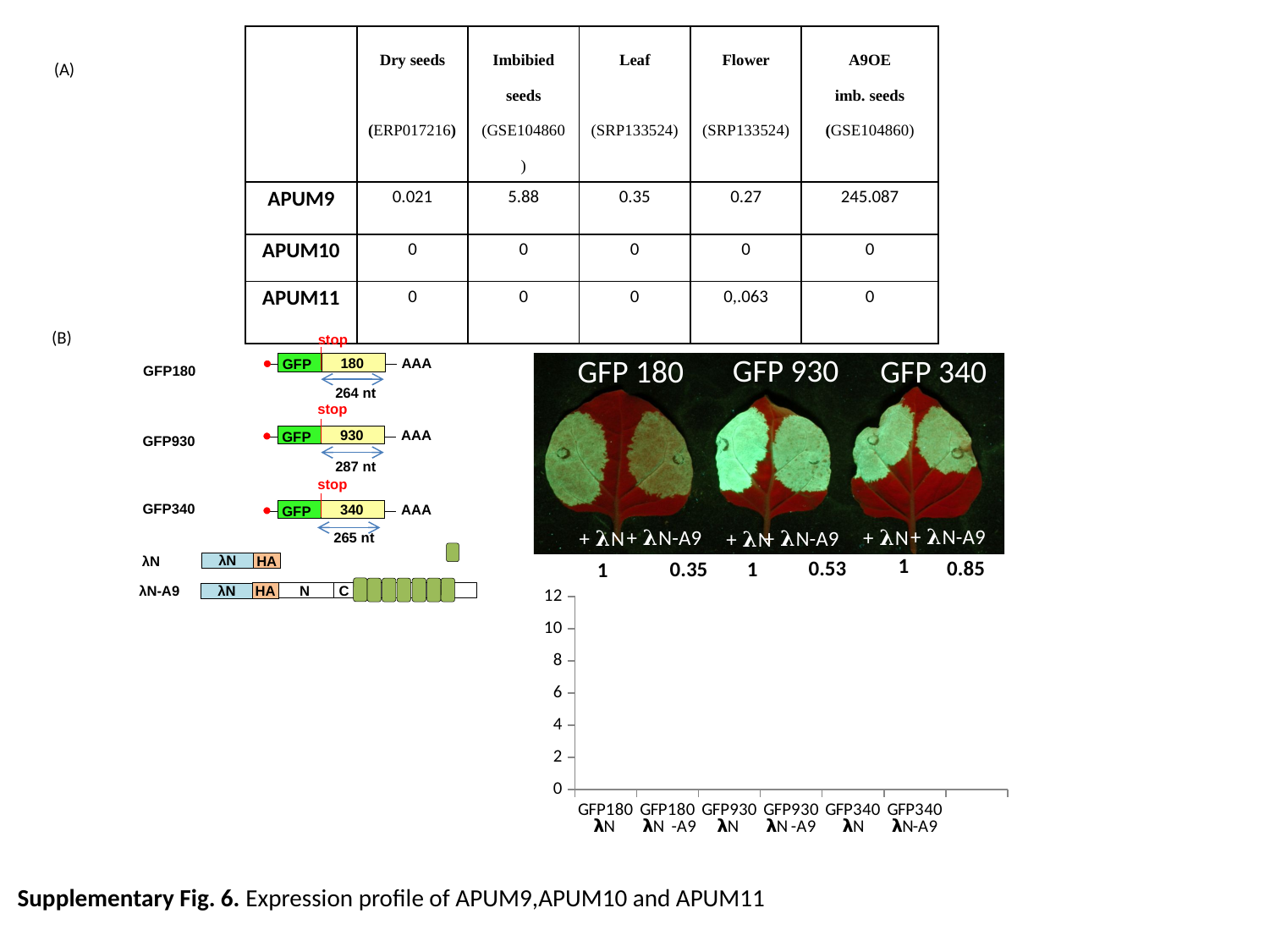

| | Dry seeds   (ERP017216) | Imbibied seeds (GSE104860) | Leaf   (SRP133524) | Flower   (SRP133524) | A9OE imb. seeds (GSE104860) |
| --- | --- | --- | --- | --- | --- |
| APUM9 | 0.021 | 5.88 | 0.35 | 0.27 | 245.087 |
| APUM10 | 0 | 0 | 0 | 0 | 0 |
| APUM11 | 0 | 0 | 0 | 0,.063 | 0 |
(A)
(B)
stop
GFP 930
GFP 180
GFP 340
+ 𝛌N-A9
+ 𝛌N
+ 𝛌N-A9
+ 𝛌N
+ 𝛌N-A9
+ 𝛌N
AAA
GFP
180
GFP180
264 nt
stop
AAA
GFP
930
GFP930
287 nt
stop
GFP340
AAA
GFP
340
265 nt
1
λN
0.53
0.85
0.35
1
1
λN
HA
N
C
λN-A9
HA
λN
### Chart
| Category | |
|---|---|
| GFP180 𝝺N | 1.0 |
| GFP180 𝝺N -A9 | 0.3836002562459961 |
| GFP930 𝝺N | 1.0 |
| GFP930 𝝺N -A9 | 0.5269288847343065 |
| GFP340 𝝺N | 1.0 |
| GFP340 𝝺N-A9 | 0.7879325643300799 |
Supplementary Fig. 6. Expression profile of APUM9,APUM10 and APUM11
