# Supplementary material for: Functional and molecular characterization of the conserved Arabidopsis PUMILIO protein, APUM9
Source: Plant Mol Biol. 2019 Mar 13;100(1):199–214. doi: 10.1007/s11103-019-00853-7 (PMC6513901; doi:10.1007/s11103-019-00853-7)
Supplement: Supplementary file 2 — Supplementary material 2 (DOCX 40 KB)—Data S1 [file 11103_2019_853_MOESM2_ESM.docx]

**Functional and molecular characterization of the conserved Arabidopsis PUMILIO protein, APUM9**

Plant Molecular Biology

Tünde Nyikó^1,2^, Andor Auber^2,^ Etienne Bucher^1^

^1^ Université d’Angers, UMR1345 Institut de Recherche en Horticulture et Semences (IRHS-INRA), 42 rue Georges Morel, 24 49071 Beaucouzé, France

^2^ Agricultural Biotechnology Institute, Szent-Györgyi Albert 4, H-2100, Gödöllő,

For correspondence: Etienne Bucher; Tel: +33 (0)2 41 22 56 99; email: [etienne.bucher@inra.fr](mailto:etienne.bucher@inra.fr)

**Table S1. Primers used for cloning:**

| **Name** | **Sequence** |
| --- | --- |
| APUM9 SpeIF | CATAACTAGTCATGGGTTTTGGAGGTTTTAATG |
| APUM9 SpeIR | CATAACTAGTTCACTTCTTCAAGATGGTCTTG |
| APUM9 Nt XhoI R | CATACTCGAG TCATGGACTCATCTTCCCATAAG |
| APUM9 Ct SpeI F | CATAACTAGTCATGAAAAGCAATAATGATCTCGTCTC |
| 5BB BamHI F | CATAGGATCCATTAAAGGTTCCTTTGTTCCC |
| 5BB XbaI R | CATATCTAGACAGAATCCAGATGCTCAAGGC |
| GFP ACC61 F | GTACATGGTACCATGAGTAAAGGAGAAGAACTT |
| GFP ACC61 R | CATAGGTACCCTATTTGTATAGTTCATCCATGCCA |
| GFPnost ACC61 R | GATCATGGTACCGGTTTGTATAGTTCATCCATGCCA |
| DCP2 BamHI F | CTGAGGATCCGAGGTATGTCAGGACTTCATCGATCTTC |
| DCP2 XbaI R | CATATCTAGAACCTCAAGAAGAGaAACCAGCTTCCATTG |
| AT1G61340 BamHI F | CAGTGCGGATCCATCAAACAACTTGGTTTCTTCAAGATTC |
| AT1G61340 XbaI R | CAGTGCTCTAGAATCTTACCAAGCCACTGTTCTTGAGAC |
| AT1G62180 nested F | GCTAAGCAAGAGCTTCAGTTAGGGAGCT |
| AT1G62180 BamHI F | CAGTGCGGATCCGTTATGAGATAGAAAATAAAAAGAGGGTGATGAAG |
| AT1G62180 XbaI R | CAGTGCTCTAGAGTTCTGCATACCTTGGTTGCGC |
| AT5G24930 BamHI F | CAGTGCGGATCCATATTGAGAGAGGGAGAGACTCTAAAATGG |
| AT5G24930 XbaI R | CAGTGCTCTAGACATAGTGATTCCGCCGCAAG |

**Table S2. T-DNA primers:**

| **Name** | **Sequence** |
| --- | --- |
| Apum9 135897SalkF | GTTAATGAAATTTGGCAGGAACTATGTGGTG |
| Apum9 135897SalkR | GTTACTAATCAAAGTCACTTCTTCAAGATGGT |
| Apum9 028481SalkF | CTGAGAACTTTTGGCCAAAAGCATAGA |
| Apum9 028481SalkR | CCACATAGTTCCTGCCAAATTTCATTAACT |
| LBA1 | TCAAACAGGATTTTCGCCTGCT |
| Gabi LBA1 | TCTCCATATTGACCATCATACTCATT |

**Table S3. qRT-PCR primers:**

| **Name** | **Sequence** |
| --- | --- |
| APUM9qF | ATAGTTCTCTGCTGAGTCCATTTC |
| APUM9qR | TGGACTCATCTTCCCATAAGAGC |
| qRT_UBQ10_F | GGCCTTGTATAATCCCTGATGAATAAG |
| qRT_UBQ10_R | AAAGAGATAACAGGAACGGAAACATAGT |
| AT1G62180qF1 | CTGATCGAACCCATTTGTCTCAGAG |
| AT1G62180qR1 | AGTTGAAGCACGAGTAACCCAAG |
| AT5G24930qF1 | GGTAACGGAAGCCGTGAAGAAG |
| AT5G24930qR1 | CTCAGCTGAAGTAACAGCAACGATC |
| AT1G01720qF1 | GGTGCCGAAGCTGCATACTACG |
| AT1G01720qR1 | CTGAACCTCGCTCGTGAACTCC |
| AT1G61340qF1 | CTGGTGATGAGATGGAGGATTCG |
| AT1G61340qR1 | CGACTCTGACCCTCTGCAACC |
| AT5G57050qF1 | CTGGCGACGGAGCTATGAAAGA |
| AT5G57050qR1 | GCGGAGATATCCACACCTGCC |
| AT4G29190qF1 | GATCGGAGAAACTCGCAGGAC |
| AT4G29190qR1 | CTCCGACGTTGTAAGCTCTTCAAG |
| GFP qF | ACA AGC AAA AGA ACG GCA TC |
| GFP qR | AAA GGG CAG ATT GTG TGG AC |
| Nb Ubiquitin qF | GCC GAC TAC AAC ATC CAG AAG G |
| Nb Ubiquitin qR | TGC AAC ACA GCG AGC TTA ACC |

**Data S1: Description of cloning**

Derivatives of the Bin61S binary vector were used both for agroinfiltration-based transient assays and for floral dip transformation (Kertész et al. 2006). Reporter genes were cloned into the binary vectors between the 35S promoter and terminator sequences. Binary vectors contain all cis sequences required for agrobacterium-mediated plant transformation or transient expression. The P14 silencing suppressor, GFP5BB (G-3’bB) reporter construct, λN, NAN, PDS (TRV-P), PDS-XRN4 (TRV-P-X4), PDS-SKI2 (TRV-P-SKI2) and PPG clones were previously described (Nyikó et al. 2009; Benkovics et al. 2011; Merai et al. 2013; Nyikó et al. 2017).

***N. benthamiana* DCP2, DCP2DN, CAF1a, CAF1aDN were provided by Daniel Silhavy prior publication:**

**DCP2DN** - Catalitically active glutamates (E-151 and E-155) within the Nudix box of the NbDCP2 enzyme were mutated to alanine, as single substitution mutations (Gunawardana et al. 2008). Analogous mutations in AtDCP2 (Arabidospis REVLEE motif mutated to RAVLEA by changing E154 to A and E 158 to A) resulted strong reductions in decapping activity.

**CAF1aDN**- contains point mutations in the conserved putative catalytic residues D33/A and E35/A that completely abolished the exonuclease activity of its Arabidopsis homolog (Liang et al. 2009).

The **λN-A9**, HA-tagged tethering construct was generated to study the effect of APUM9 binding on the expression of target transcripts. The coding region of Arabidopsis APUM9 gene were PCR amplified with the APUM9 SpeI F/ APUM9 SpeI R primer pairs. The SpeI digested PCR product was cloned in frame with a λN-HA tag, into a properly digested, dephosphorylated BinλNHA vector.

The Ha tagged **A9** construct were made by PCR using the APUM9 SpeI F/ APUM9 SpeI R primer pairs. The SpeI digested PCR product was cloned into a properly digested, dephosphorylated Bin HA vector in frame with a HA tag.

The **λN-A9Nt, λN-A9Ct**, HA-tagged clones were used to map whether the N-, or the C-terminal domain is required for APUM9 tethering-induced RNA degradation. The N-, and the C-terminal part of APUM9 were PCR amplified using APUM9 SpeI F/ APUM9 Nt XhoI R and the APUM9 Ct SpeI F/ APUM9 SpeI R primer pairs, respectively. The N-terminal part (738 nt from start) was inserted into a SpeI/SalI digested BinλNHA vector, in frame with λN-HA tag. The SpeI digested C-terminal part (983nt) was cloned in frame with λN-HA into a SpeI digested, dephosphorylated BinλNHA vector. XhoI and SalI cut compatible cohesive ends.

**GUS-5BB** reporter was used to measure the GUS activity and GUS mRNA level after APUM9 tethering. To create GUS5BB construct, the 5BoxB (BB) tethering target sequence, that contains five direct repeats of BB, was PCR amplified from GFP5BB plasmid with 5BB BamHI F/5BB XbaI R primers, and then it was cloned into the 3’-UTR region of Bin61SGUS.

**GFP** control construct and **GFP-DCP2** and **GFP-APUM9** fusion vectors were generated to identify APUM9 protein partners by immunoprecipitation. To obtain GFP control construct, PCR amplified (GFP ACC61 F/ GFP ACC61 R from GFP-5BB plasmid template), ACC65I digested GFP PCR fragment were cloned into an ACC65I cleaved, dephosphorylated Bin61S vector. N. benthamiana DCP2 and Arabidopsis APUM9 genes were PCR amplified from DCP2 and λN-A9 plasmid templates with the DCP2 BamHI F/DCP2 XbaI R and APUM9 SpeI F/APUM9 SpeI R primer pairs respectively. Digested DCP2 and APUM9 PCR products were inserted into the properly digested GFPnostop vector (a GFP reporter construct lacking the stop codon: GFP ACC61 F/ GFPnostop ACC61 R PCR fragment cloned into Bin61S vector), in frame with GFP.

**A9-OE** was constructed to assess the effect of APUM9 overexpression in plant development and in heat tolerance and to recognize the potential APUM9 target transcripts. PCR amplified and SpeI digested APUM9 fragment was cloned into a XbaI digested, dephosphorylated Bin61S binary vector containing the constitutive 35S promoter of Cauliflower mosaic virus (CMV). SpeI and XbaI cut compatible cohesive ends.

**GFP180, GFP340, GFP930** constructs were generated to investigate whether similar to other eukaryotic PUF proteins, APUM9 acts through binding to the 3'UTR of their target mRNAs. The terminator regions (after the stop codon to +200 relative to the annotated polyadenylation site) of the respective genes (AT1G62180, AT5G24930, AT1G61340) were PCR amplified from *Arabidopsis* genomic DNA with the AT1G61340BamHIF/AT1G61340XbaIR, AT1G62180BamHIF/ AT1G62180XbaIR, AT5G24930BamHI F/AT5G24930XbaI R primer pairs, respectively. One nested PCR were used to specifically amplify AT1G62180. BamHI-XbaI digested PCR fragments were then cloned after the stop codon of BamHI-XbaI digested GFP reporter construct.

**Supplementary Figure legends**

**Supplementary Fig.1 Conserved RNA-binding domain of APUM9 displays high similarity to the yeast PUF4 protein in key amino acid positions.**

Alignment of the nucleotide binding residues of human and yeast Pumilio proteins with the corresponding residues in the Arabidopsis APUM1, APUM9 and the putative N. Benthamiana PUMILIO homologs (NbPUMILIO12, NbPUMILIO11, NbPUMILIO9) that shows high level of sequence similarity to Arabidopsis APUM9. Note that NbPUMILIO9 sequence is not complete. Rectangles highlight the strongly conserved amino acid region inside the repeates.

**Supplementary Fig.2 APUM9 without λN tethering could not bind to GFP reporter mRNA. a** Schematic, non-proportional representation of the constructs used for agroinfiltration. **b** To further confirm that mRNA degradation was activated by APUM9 tethering and not by an unspecific interaction between APUM9 RBD and GFP5BB reporter, GFP5BB reporter plus P14 silencing suppressor were co-expressed in *N. Benthamiana* leaves with full length λN-A9 positive control or λN and A9 negative controls and examined as described at Figure 1c-d. Photo and RNA-, protein samples were taken at 3 days post infiltration (d.p.i). Photo was taken under UV light, thus the non-infiltrated parts of the leaf are red due to the autofluorescence of chlorophyll, while the GFP expressing agroinfiltrated patches show green fluorescence. **c** RNA gel blot was hybridized with GFPand P14 probes and the reporter mRNA (GFPprobe) was normalized to the corresponding P14 signal (GFPP14 signal). **d** GFP Western blot further confirmed that GFP5BB expression is significantly lower in λN-A9 co-infiltrated samples compared to the λN and A9 controls. GFP protein signals were normalized to the corresponding comassie blue stained total protein level. **e** The expression of HA tagged APUM9 construct was confirmed by western blot.

**Supplementary Fig.3 APUM9 tethering into the 3’UTR induces rapid mRNA destabilization. a** Schematic, non-proportional representation of the constructs used for tethering assay. **b** To further confirm that APUM9 binding activates mRNA degradation, GUS5BB reporter plus P14 silencing suppressor were co-expressed in *N. Benthamiana* leaves with full length, N-, or C- terminal APUM9 tethering constructs (λN-A9, λN-A9Nt, λN-A9Ct) and examined as described at Figure 1c-d. RNA gel blot was hybridized with GUS and P14 probes and the reporter mRNA (GUS probe) was normalized to the corresponding P14 signal (GUS*/*P14 signal). **c** Total protein lysates were prepared at 3 d.p.i. to measure GUS and NAN activity. NAN is a compatible functionally equivalent reporter partner of GUS that possesses similar kinetic and stability properties to GUS, and showed optimal activity in GUS buffer. GUS activity of GUS5BB reporter in each sample was normalized to the corresponding NAN control signal. Mean values were calculated from three independent samples (n=3) and compared. The GUS*/*NAN activity ratios of λN-A9, λN-A9Nt, λN-A9Ct co-infiltrated samples were compared to GUS5BB+ λN control sample.

**Supplementary Fig.4 APUM9 has only minor role in plant heat response. a** qRT-PCR analysis of APUM9 transcript levels from different tissues. The expression values were normalized using UBIQUITIN as control. n = 3 biological replicates, error bars represent standard deviations. **b** Fresh weight of Col-0 wild-type and A9-OE Arabidopsis plants were measured. Pictures show the analyzed 1 week old seedlings grown on MS media, at constant 28°C, under 16/8 h light/dark conditions. Bars represent the mean of fresh weight measurement from 15 seedlings. Error bars indicate standard deviation (SD) of the mean. **c** Heat shock treated 1 week old Col-0 wild-type and A9-OE Arabidopsis plants died 1 week after heat shock.

**Supplementary Fig.5 Predicted role of APUM9 in seed dormancy regulation. a** ABA is involved in the maintenance of seed dormancy in dry seeds and also inhibits the transition from embryonic to germination growth. **b** We propose that strong epigenetic repression of APUM9 is released during seed imbibition and APUM9 might be involved in the reprogramming of ABA genes thus switch ABA signaling in the favor of seed germination.

**Supplementary Fig. 6. Expression profile of APUM9,APUM10 and APUM11.** **a** APUM9, APUM10 and APUM11 expression were compared using our GSE104860 RNA seq and publicly available transcriptome data (ERP017216 and SRP133524). The values are given in FPKM (Fragment per kilobase per million read). **b** To test the effect of APUM9 binding on endogenous target mRNA, 3’UTR regions of APUM9 downregulated transcripts were cloned after GFP reporter and co-expressed in *Nicotiana benthamiana* leaves with full-length APUM9 constructs (λN-A9). The length of 3’UTRs are shown below the constructs. As control, GFP constructs were infiltrated with λN. Photo were taken at 3 days post infiltration (d.p.i) under UV light. The intensity of GFP fluorescence was measured by ImageJ scientific image analysis software. qRT-PCR analysis of GFP transcript levels from infiltrated leaf patches were measured. The expression values were normalized using UBIQUITIN as control. n = 3 biological replicates, error bars represent standard deviations.

**SUPPLEMENTARY REFERENCES**

Benkovics AH, Nyikó T, Mérai Z, Silhavy D, Bisztray GD (2011) Functional analysis of the grapevine paralogs of the SMG7 NMD factor using a heterolog VIGS-based gene depletion-complementation system. Plant Mol Biol 75:277–90 . doi: 10.1007/s11103-010-9726-0

Gunawardana D, Cheng HC, Gayler KR (2008) Identification of functional domains in Arabidopsis thaliana mRNA decapping enzyme (AtDcp2). Nucleic Acids Res 36:203–216 . doi: 10.1093/nar/gkm1002

Kertész S, Kerényi Z, Mérai Z, Bartos I, Pálfy T, Barta E, Silhavy D (2006) Both introns and long 3’-UTRs operate as cis-acting elements to trigger nonsense-mediated decay in plants. Nucleic Acids Res 34:6147–57 . doi: 10.1093/nar/gkl737

Liang W, Li C, Liu F, Jiang H, Li S, Sun J, Wu X, Li C (2009) The Arabidopsis homologs of CCR4-associated factor 1 show mRNA deadenylation activity and play a role in plant defence responses. Cell Res 19:307–316 . doi: 10.1038/cr.2008.317

Merai Z, Benkovics AH, Nyiko T, Debreczeny M, Hiripi L, Kerenyi Z, Kondorosi E, Silhavy D (2013) The late steps of plant nonsense-mediated mRNA decay. Plant J 73:50–62 . doi: 10.1111/tpj.12015

Nyikó T, Auber A, Szabadkai L, Benkovics A, Auth M, Mérai Z, Kerényi Z, Dinnyés A, Nagy F, Silhavy D (2017) Expression of the eRF1 translation termination factor is controlled by an autoregulatory circuit involving readthrough and nonsense-mediated decay in plants. Nucleic Acids Res 45: . doi: 10.1093/nar/gkw1303

Nyikó T, Sonkoly B, Mérai Z, Benkovics AH, Silhavy D (2009) Plant upstream ORFs can trigger nonsense-mediated mRNA decay in a size-dependent manner. Plant Mol Biol 71:367–78 . doi: 10.1007/s11103-009-9528-4
